# Optimization of the Extraction Conditions and Evaluation of Bioactivities of the Phenolic Enrichment From *Pandanus amaryllifolius* Leaves

**DOI:** 10.1155/jamc/5256388

**Published:** 2025-05-07

**Authors:** Do Hoang Giang, Bui Thi Nhat Le, Nguyen Thi Thu Minh, Nguyen Thi Thu Thuy, Nguyen Hai Dang, Hoang Le Tuan Anh, Nguyen Ngoc Tung, Nguyen Tien Dat

**Affiliations:** ^1^University of Science and Technology of Hanoi, Vietnam Academy of Science and Technology (VAST), 18-Hoang Quoc Viet, Cau Giay, Hanoi 10000, Vietnam; ^2^Center for High Technology Research and Development, VAST, 18-Hoang Quoc Viet, Cau Giay, Hanoi 10000, Vietnam; ^3^Joint Vietnam-Russia Tropical Science and Technology Research Center, Nguyen Van Huyen, Cau Giay, Hanoi 10000, Vietnam

**Keywords:** antioxidant, Box–Behnken, *Pandanus amaryllifolius*, phenolic

## Abstract

This study investigates the optimal conditions to enrich the phenolic content of the extract from *Pandanus amaryllifolius* leaves and evaluates the bioactivities of this enrichment. The phenolic enrichment was prepared under optimized conditions using the response surface methodology (RSM) with the Box–Behnken design. The antioxidant properties were assessed using DPPH and hydroxyl radical scavenging assays, while the NO production inhibition was measured in LPS-stimulated RAW 264.7 macrophage cells. Results indicated that the phenolic enrichment showed potent antioxidant activity comparable to ascorbic acid and catechin and significantly higher NO inhibition than the separated nonalkaloid and alkaloid fractions. The study also highlights the synergistic effect of phenolic and alkaloid compounds on the antioxidants and anti-inflammatory activities of the phenolic enrichment from *Pandanus amaryllifolius* leaves.

## 1. Introduction

*Pandanus amaryllifolius* Roxb is an evergreen tree with fragrantly scented leaves. The plant is cultivated for its leaves in gardens in Vietnam, Indonesia, Malaysia, Thailand, New Guinea, Sri Lanka, and the Philippines [1, 2]. Phytochemical studies reported that alkaloids were the major secondary metabolites in *P. amaryllifolius* with considerable anti-inflammatory and anticancer effects [2–6]. Besides, flavonoids, coumaroyl malate, and coumarin derivatives, together with terpenoids and other organic compounds, were determined from leaves of *P. amaryllifolius* [7–12]. While these findings highlight the diverse chemical profile of *P. amaryllifolius*, detailed quantitative data on the composition of these compounds remain limited and warrant further investigation. Furthermore, variations in the reported antioxidant effects of alkaloids across studies have raised questions about the consistency of these results [9–11]. Antioxidants, particularly those derived from plant sources, play a crucial role in mitigating oxidative stress, which is linked to various chronic diseases such as cancer, cardiovascular diseases, and neurodegenerative disorders. Plant-based antioxidants offer advantages over synthetic counterparts due to their biocompatibility, lower toxicity, and additional bioactive properties. In this context, the optimization of extraction processes becomes essential to maximize the yield and efficacy of phytoconstituents.

Conventional extraction methods often suffer from limitations such as low selectivity, high solvent consumption, and extended processing times [13]. To overcome these challenges, the response surface methodology (RSM) with the Box–Behnken design (BBD) was employed for optimizing the extraction of bioactive products from natural sources [14]. The BBD was chosen for its efficiency in evaluating interactions among variables with fewer experimental runs than traditional methods [13, 14]. Parameters such as solvent concentration, extraction time, and temperature were systematically optimized to enrich the phenolic content, significantly improving the extraction yield.

Previously, we investigated alkaloids from the aerial part of *P. amaryllifolius* from Vietnam and evaluated their anti-inflammatory activity [15]. In the current study, we continuously determined and elucidated the structures of phenolics from the nonalkaloid fraction of the samples. The extracting process was optimized to enrich the phenolic content using the RSM with BBD. Moreover, the antioxidant and NO production inhibitory effects of the fractions and compounds were investigated.

## 2. Materials and Methods

### 2.1. Plant Materials

Leaves of *P. amaryllifolius* were collected at Tam Dao, Vinh Phuc Province, Vietnam, in April 2021 and identified by Dr. Bui Van Thanh, Institute of Ecology and Biological Resources, Vietnam Academy of Sciences and Technology (VAST). A voucher specimen (NCCG 210213) was deposited at the Department of Agro-Pharmaceutical Research, Center for High Technology Research and Development, VAST. The collected sample was cleaned, dried at 50°C in the oven, powdered, and preserved at −20°C for further experiments.

### 2.2. General

ESI-MS was measured by a Thermo LCQ Fleet system. NMR spectra were recorded on a Bruker AVANCE NEO 600-MHz spectrometer with tetramethylsilane (TMS) as an internal standard. Column chromatography (CC) was carried out using Diaion HP-20 resin (0.25–0.85 mm, Mitsubishi Chemical Corp., Japan), silica gel 60 (70–230 mesh, Merck, Germany), or RP-C18 resin (150 μm, YMC, Japan). HPLC analysis and prep-HPLC were conducted on a Thermo Ultimate 3000 HPLC-DAD and an Agilent 1100 system. The extraction was processed in a shaking water bath (Daihan Scientific).

### 2.3. Total Phenolic Assay

The total phenolic contents (TPC) of the samples were evaluated by the Folin–Ciocalteu method [16]. In brief, 0.1 mL of each solution of *P. amaryllifolius* leaves' extract was mixed with 0.9 mL of Folin–Ciocalteu 10% and 1.0 mL of Na_2_CO_3_ 6% solution. Next, the mixture was incubated at 40°C for 15 min, and then the absorbance was measured at 760 nm. Gallic acid, which was processed under the same condition, was used as the standard compound for the calibration curve of the measurement.

### 2.4. Preliminary Single-Factor Experiments

Ranges of the extraction factors, such as temperature, ethanol concentration, and time, were evaluated from the following experiments. Firstly, the effect of extraction temperature on the TPC of the extracts was investigated by extracting the material in ethanol 96% at 30°C–80°C for 180 min. Next, the impact of the ethanol concentration on the TPC values was determined by extracting the *P. amaryllifolius* leaves in ethanol 0%–96% for 180 min at 60°C. Lastly, the influence of extraction time on TPC was evaluated by extracting the material in ethanol 96% at 60°C for 60–360 min. In each experiment, 5 g of *P. amaryllifolius* leaves was extracted in 150 mL of solvent, maintaining a fixed ratio of leaf weight to solvent volume at 1:30 (g/mL).

### 2.5. RSM

The RSM applying the BBD was utilized to design the experiments to optimize the condition to enrich phenolics into the extract of *P. amaryllifolius* leaves. The design was conducted on the Design-Expert 12.0 software (Stat-Ease, Inc., Minneapolis, US). Extraction temperature (°C, *X*_1_), ethanol concentration (%, *X*_2_), and extraction time (minute, *X*_3_) were selected as independent factors, while TPCs were selected as the responses. From preliminary single-factor experiments, ethanol 0% (distilled water), 48%, and 96% were used as the solvents, whereas the temperature ranged from 30°C to 80°C; meanwhile, the extraction time varied between 60 and 300 min. Each experiment was conducted in triplicate and the mean of the response (TPC) was used for further calculation. The levels of the variables in the experimental design are shown in Table 1.

### 2.6. Extraction and Isolation

The phenolic enrichment from the optimal extracting condition was further isolated by chromatographic methods. 200 g of the enrichment was acidified with 1 N HCl solution to pH 2–3 and successively partitioned with ethyl acetate (EtOAc) (2 L × 4 times). The organic layer was separated and completely evaporated to afford nonalkaloid extract (62.4 g). The water layer which contained alkaloids was basified by 1 N NaOH to pH 9–10 and was extracted with CH_2_Cl_2_ (4 × 2 L). The CH_2_Cl_2_ layers were combined to collect the alkaloid fraction (22.6 g). The alkaloid fraction was analyzed and compared to the reported compounds in our in-house library [15] using the HPLC-DAD system to identify its composition.

The nonalkaloid extract was subjected to a Diaion HP-20 CC and then the column was washed with water, followed by MeOH 30% and 100% to obtain M30W (4.5 g) and M100W (13.6 g) fractions, respectively. The fraction M30W was loaded on a silica gel column with gradient mixtures of CH_2_Cl_2_–MeOH (20/1-1/1, v/v) to afford four subfractions (W1–W4). Fraction W1 (105 mg) was separated by preparative HPLC (120 min, 30%–60% MeOH in H_2_O) to yield the compound **Pam1** (11.2 mg). The fraction M100W was subjected to a silica gel column eluted with gradient mixtures of CH_2_Cl_2_–MeOH (50/1-1/1, v/v) to afford 10 subfractions (M1–M10). Fraction M6 (91 mg) was separated by preparative HPLC (120 min, 40%–100% MeOH in H_2_O) to yield the compounds **Pam2** (15.5 mg) and **Pam3** (6.8 mg). Fraction M4 (304 mg) was separated by preparative HPLC (120 min, 30%–80% MeOH in H_2_O) to yield the compounds **Pam4** (4.1 mg) and **Pam5** (6.6 mg). Fraction M8 (178 mg) was chromatographed on a silica gel CC eluted with CH_2_Cl_2_–acetone (5/1, v/v) to afford compounds **Pam6** (3.1 mg) and **Pam7** (5.6 mg). Fraction M5 (91 mg) was chromatographed on a silica gel CC eluted with CH_2_Cl_2_–methanol (9/1, v/v) to afford compound **Pam8** (5.6 mg).

Pinoresinol 4*-O-β-D*-glucoside **(Pam1)**: white amorphous powder, ESI-MS: *m/z* 521 [M + H]^+^; *m/z* 1041 [2M + H]^+^; molecular formula: C26H32O11; ^1^H NMR (CD_3_OD, 500 MHz): *δ* 7.05 (1H, d, *J = *2.0 Hz, H-2), 7.17 (1H, d, *J = *8.5 Hz, H-5), 6.94 (2H, dd, *J = *8.5; 2.0 Hz, H-6), 4.78 (1H, d, *J = *4.5 Hz, H-7), 3.15 (2H, m, H-8), 3.72 (2H, dd, *J = *9.0; 4.5 Hz, H-9a), 4.26 (2H, dd, *J = *9.0; 2.0 Hz, H-9b), 6.97 (1H, d, *J = *2.0 Hz, H-2′), 6.79 (1H, d, *J = *8.5 Hz, H-5′), 6.94 (2H, dd, *J = *8.5; 2.0 Hz, H-6′), 4.73 (1H, d, *J = *4.5 Hz, H-7′), 3.15 (2H, m, H-8′), 3.72 (2H, dd, *J = *9.0; 4.5 Hz, H-9′a), 4.26 (2H, dd, *J = *9.0; 2.0 Hz, H-9′b), 3.88 (3H, s, 3*-O-*CH_3_), 3.89 (3H, s, 3′*-O-*CH_3_), 4.89 (1H, d, *J = *7.5 Hz, H-1″), 3.44 (1H, m, H-2″), 3.42 (1H, m, H-3″), 3.30 (1H, m, H-4″), 3.40 (1H, m, H-5″), 3.46–3.53 (2H, m, H-6″); ^13^C NMR (CD_3_OD, 125 MHz): *δ* 137.5 (C-1), 111.0 (C-2), 151.0 (C-3), 147.3 (C-4), 118.1 (C-5), 119.8 (C-6), 87.4 (C-7), 55.35 (C-8), 72.7 (C-9), 133.8 (C-1′), 111.7 (C-2′), 149.1 (C-3′), 147.5 (C-4′), 116.11 (C-5′), 119.8 (C-6′), 87.1 (C-7′), 55.54 (C-8′), 72.7 (C-9′), 56.5 (3*-O-*CH_3_), 56.8 (3′*-O-*CH_3_), 102.9 (C-1″), 74.9 (C-2″), 77.9 (C-3″), 71.4 (C-4″), 78.2 (C-5″), 62.5 (C-6″).

Pinoresinol **(Pam2)**: Yellow oil; ESI-MS: *m/z* 359 [M + H]^+^, *m/z* 341 [M + H-H_2_O]^+^, *m/z* 739 [2M + Na]^+^; molecular formula: C_20_H_22_O_6_ (358 g/mol); ^1^H NMR (CDCl_3_, 500 MHz): *δ* 7.0 (2H, brs, H-2, 2′), 6.89 (2H, d, *J = *8.0 Hz, H-5, 5′), 6.82 (2H, dd, *J = *8.0; 2.0 Hz, H-6, 6′), 4.73 (2H, d, *J = *4.0 Hz, H-7, 7′), 3.11 (2H, m, H-8, 8′), 3.88 (2H, dd, *J = *9.0; 4.0 Hz, H-9, 9′), 4.26 (2H. dd. *J = *9.0; 6.5 Hz, H-9, 9′), 3.91 (3H, s, 3*-O-*CH_3_), 3.89 (3H, s, 3′*-O-*CH_3_); ^13^C NMR (CDCl_3_, 125 MHz): *δ* 133.1 (C-1, 1′), 108.8 (C-2, 2′), 146.9 (C-3, 3′), 145.4 (C-4, 4′), 114.4 (C-5, 5′), 119.1 (C-6, 6′), 86.0 (C-7, 7′), 54.3 (C-8, 8′), 71.6 (C-9, 9′), 56.1 (3*-O-*CH_3_), 56.1 (3′*-O-*CH_3_).

Pinoresinol monomethyl ether **(Pam3)**: pale yellow oil; ESI-MS: *m/z* 373 [M + H]^+^, *m/z* 395 [M+Na]^+^; molecular formula: C_21_H_24_O_6_ (372 g/mol); ^1^H NMR (CDCl_3_, 500 MHz): *δ* 6.90 (1H, d, *J = *2.0 Hz, H-2), 6.89 (1H, d, *J = *9.0 Hz, H-5), 6.83 (1H, dd, *J = *9.0; 2.0 Hz, H-6), 4.75 (1H, d, *J = *4.5 Hz, H-7), 3.31 (1H, m, H-8), 4.25 (2H, m, H-9), 6.90 (1H, dd, *J = *6.5; 1.5 Hz, H-2′), 6.85 (1H, d, *J = *9.0 Hz, H-5′), 6.87 (1H, dd, *J = *9.0, 2.0 Hz, H-6′), 4.75 (1H, d, *J = *4.5 Hz, H-7′), 3.31 (1H, m, H-8′), 3.87 (2H, m, H-9′), 3.89 (3H, s, 3*-O-*CH_3_), 3.90 (3H, s, 3′*-O-*CH_3_), 3.91 (3H, s, 4′*-O-*CH_3_),; ^13^C NMR (CDCl_3_, 125 MHz): *δ* 132.9 (C-1), 108.7 (C-2), 148.7 (C-3), 145.3 (C-4), 114.3 (C-5), 119 (C-6), 85.8 (C-7), 54.2 (C-8), 71.7 (C-9), 133.6 (C-1′), 109.3 (C-2′), 149.3 (C-3′), 146.8 (C-4′), 111.1 (C-5′), 118.3 (C-6′), 85.8 (C-7′), 54.2 (C-8′), 71.7 (C-9′), 56.0 (3*-O-*CH_3_), 56.0 (3′*-O-*CH3), 56.0 (4′*-O-*CH_3_).

Methyl 4-hydroxybenzoate **(Pam4)**: *m/z* 153 [M + H]^+^; ^1^H NMR (DMSO-*d*_6_, 600 MHz): *δ* 7.81 (1H, d, *J = *6.6 Hz, H-2,6), 6.84 (1H, d, *J = *6.6 Hz, H-3,5), 3.78 (3H, s, OCH_3_). ^13^C NMR (DMSO-*d*_6_, 125 MHz): *δ* 120.2 (C-1), 131.3 (C-2), 115.3 (C-3), 161.9 (C-4), 115.3 (C-5), 131.3 (C-6), 166.0 (C-7), 51.6 (7-OCH_3_).

3,4-Dihydroxyl benzoate methyl **(Pam5)**: *m/z* 169 [M + H]^+^; ^1^H NMR (DMSO-*d*_6_, 600 MHz): *δ* 7.42 (1H, d, *J = *1.8 Hz, H-2), 6.83 (1H, d, *J = *7.8 Hz, H-5), 7.44 (1H, dd, *J = *1.8, 7.8 Hz, H-6), 3.80 (3H, s, OCH_3_). ^13^C NMR (DMSO-*d*_6_, 125 MHz): *δ* 121.6 (C-1), 123.4 (C-2), 151.1 (C-3), 163.2 (C-4), 112.7 (C-5), 115 (C-6), 167.1 (C-7), 55.5 (7-OCH_3_).

4-Hydroxy benzoic acid **(Pam6)**: *m/z* 139 [M + H]^+^; ^1^H NMR (DMSO-*d*_6_, 600 MHz): *δ* 7.77 (1H, d, *J = *9.0 Hz, H-2,6), 6.80 (1H, d, *J = *9.0 Hz, H-3,5). ^13^C NMR (DMSO-*d*_6_, 125 MHz): *δ* 121.3 (C-1), 131.6 (C-2), 115.5 (C-3), 161.5 (C-4), 115.5 (C-5), 131.6 (C-6), 167.1 (C-7).

Methyl 4-hydroxy-3-methoxybenzoate **(Pam7)**: *m/z* 183 [M + H]^+^; ^1^H NMR (CD_3_OD, 600 MHz): *δ* 7.54 (1H, d, *J = *1.8 Hz, H-2,6), 6.83 (1H, d, *J = *8.4 Hz, H-5), 7.54 (1H, dd, *J = *8.4, 1.8 Hz, H-2,6), 3.87 (3H, s, OCH_3_), 3.90 (3H, s, OCH_3_). ^13^C NMR (CD_3_OD, 125 MHz): *δ* 121.6 (C-1), 125.2 (C-2), 149.2 (C-3), 154.4 (C-4), 113.5 (C-5), 116.3 (C-6), 168.9 (C-7), 52.3 (3-OCH_3_), 56.4 (7-OCH_3_).

Methyl syringate **(Pam8)**: *m/z* 213 [M + H]^+^; ^1^H NMR (CD_3_OD, 600 MHz): *δ* 7.34 (2H, s, H-2,6), 3.89 (6H, s, 3,5-OCH_3_), 3.89 (3H, s, 7-OCH_3_). ^13^C NMR (CD_3_OD, 125 MHz): *δ* 121.37 (C-1), 108.1 (C-2), 148.94 (C-3), 141.94 (C-4), 148.94 (C-5), 108.1 (C-6), 168.63 (C-7), 56.8 (3,5-OCH_3_), 52.5 (7-OCH_3_).

### 2.7. Antioxidant and NO Production Inhibition Assay

The antioxidant activities of the phenolic enrichment were evaluated by DPPH and hydroxyl radicals scavenging assays using previously established methods [17, 18]. For DPPH free radical scavenging effect, 100 μL of each sample was combined with 1900 μL of DPPH in methanol and incubated in the dark at 37°C for 20 min. The absorbance was then measured at 517 nm, with ascorbic acid as the positive control. For the hydroxyl radicals scavenging assay, 200 μL aliquot of the test sample was combined with 400 μL of 50 mM phosphate buffer (pH 7.8), 400 μL of 2.8 mM deoxyribose, and 400 μL of 500 μM Fe(NH_4_)_2_(SO_4_)_2_ and then incubated at 37°C for 1 h. The reaction was stopped by adding 1000 μL of 10% (w/v) trichloroacetic acid and 1000 μL of 1% (w/v) thiobarbituric acid, followed by heating the mixture in a water bath at boiling temperature for 15 min. The absorbance of the resulting solution was read at 532 nm, using catechin as a positive control.

The impact of samples on NO production in LPS-stimulated RAW 264.7 macrophage cells was assessed using the Griess reaction [19]. Cells were seeded in 96-well plates at a density of 0.5 × 10^5^ cells per well and incubated in a humidified chamber at 37°C with 5% CO_2_ for 22 h. After incubation, samples at concentrations ranging from 3 to 25 μg/mL were added, followed by the addition of 0.1 mg/mL LPS (Sigma-Aldrich, USA) after 30 min. The cells were then incubated for an additional 24 h. Subsequently, 100 μL of the culture supernatant was transferred to a new 96-well plate and combined with 100 μL of the Griess reagent. The absorbance of the reaction mixture was measured at 570 nm using an iMark microplate reader (Bio-Rad, USA). The remaining cells in the original 96-well plate were used for the 3-(4,5-dimethylthiazol-2-yl)-2,5-diphenyltetrazolium bromide (MTT) assay to evaluate cell viability, based on the reduction of MTT by mitochondrial dehydrogenases in viable cells, thus estimating the number of live cells. Cardamonin, a known NO production inhibitor, was used as a positive control.

## 3. Results and Discussion

### 3.1. The Process Range Conditions for the Extraction

**Figure 1:**
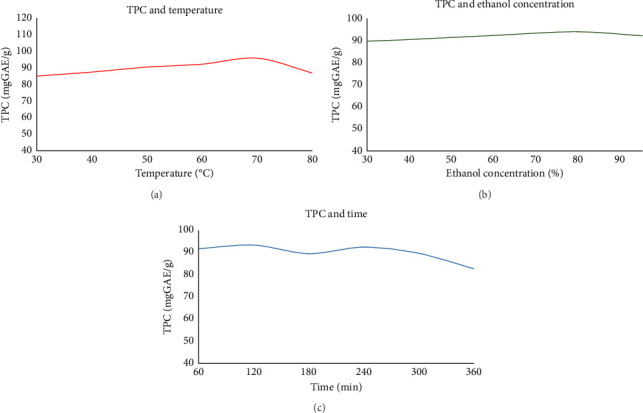
Effect of extraction temperature (a), ethanol concentration (b), and extraction time (c) on total phenolic content of extracts from *P. amaryllifolius* leaves.

The impact of temperature, ethanol concentration, and extraction time on the TPC of *P. amaryllifolius* leaf extracts was systematically investigated. The data from the experimental design, summarized in Table S1 and Figure 1, reveal distinct trends associated with each parameter, leading to the identification of optimal ranges for phenolic extraction.

The TPC was positively correlated with temperature within the range of 30°C–80°C, with the highest TPC (95.84 mg GAE/g) observed at 70°C, and slightly decreased to 86.93 mg GAE/g at 80°C. These suggested that higher temperatures may cause degradation of phenolic compounds or excessive evaporation of ethanol. For practical purposes and to align with the experimental environment's ambient conditions, temperatures below 30°C were deemed unsuitable. These findings confirm that the temperature range for maximizing phenolic extraction should lie between 30°C and 80°C.

Meanwhile, the ethanol concentration influenced the TPC, with relatively small differences observed across the tested range. The TPC increased steadily with rising ethanol concentrations, from 80.38 mg GAE/g at 0% ethanol (distilled water) to 93.93 mg GAE/g at 80% ethanol, after which it slightly decreased to 92.12 mg GAE/g at 96% ethanol. While 80% ethanol yielded the highest TPC, the variations between concentrations above 40% were not substantial. This result highlights the need to optimize ethanol concentration further within the range of 0%–96% to identify the precise conditions that balance extraction efficiency and solvent consumption.

The extraction time also had a noticeable impact on TPC, with values generally increasing within the range of 60–240 min. The highest TPC (93.37 mg GAE/g) was observed at 120 min, indicating this duration was particularly effective for phenolic extraction. While longer times, such as 240 min, still yielded relatively high TPC (92.46 mg GAE/g), a gradual decline was observed beyond this point, with 89.67 mg GAE/g at 300 min and 82.67 mg GAE/g at 360 min. This reduction could be attributed to the potential degradation of phenolic compounds or decreased solvent efficiency over prolonged periods. Shorter durations, such as 60 min, also provided a reasonably high TPC (91.73 mg GAE/g), suggesting that the extraction process reaches a significant level of efficiency early on. However, extending the time to 240 min ensures thorough extraction, particularly for phenolic compounds that may require more time to diffuse into the solvent. Based on these findings, the optimal time range for extraction is 60–300 min, providing flexibility to balance efficiency and resource consumption while minimizing the risk of phenolic degradation.

### 3.2. Optimizing the Extracting Condition to Prepare the Phenolic Enrichment From *P. amaryllifolius* Leaves.

The RSM with BBD was applied to determine the optimal condition for the extraction of phenolic from *P. amaryllifolius* leaves. The extraction design variables' effect on the TPC values is given in Table 2.

The modified quadratic models for the estimation of polyphenol content (TPC) in terms of extracting temperature (*X*_1_), ethanol concentration (*X*_2_), and extracting time (*X*_3_) are shown below:

(1) $$\begin{array}{c} \text{TPC}=-13.700711+2.12296X_{1}+0.956150X_{2}+0.159595X_{3}-0.007927X_{1}X_{2}-0.001509X_{1}X_{3}-0.000261X_{2}X_{3}-0.012019X_{1}^{2}-0.004918X_{2}^{2}-0.000149X_{3}^{2}+0.000032X_{1}X_{2}^{2}. \end{array}$$

**Figure 2:**
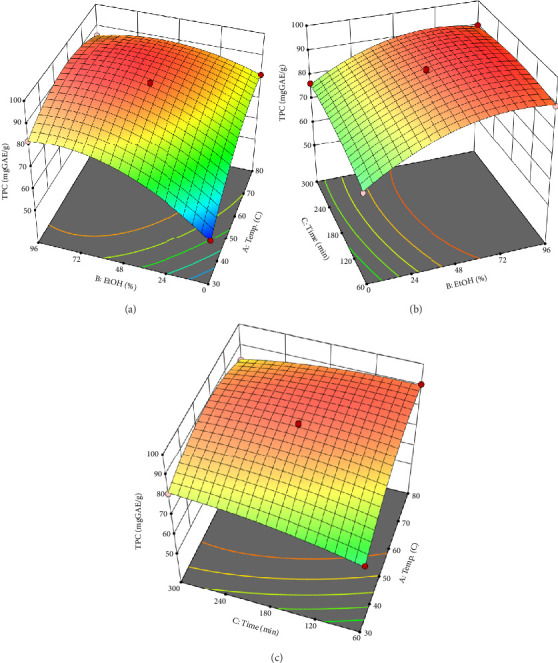
Response surfaces between (a) temperature and ethanol concentration, (b) ethanol concentration and time in response, and (c) time and temperature to total phenolic contents of the *P. amaryllifolius* extracts.

The analysis of variance illustrated the model F value at 90.26 with *p* < 0.0001 which implied that the model was highly significant. The lack of fit was insignificant (*p* > 0.05), indicating that the model fit the analytical results. The model, adjusted, and predicted *R*^2^ at 0.9912, 0.9802, and 0.9437, respectively, implied that the model could be validated for use in the investigated ranges. The response surface plots of ethanol concentration–extracting temperature, ethanol concentration–extracting time, and extracting time–temperature are shown in Figure 2.

As can be seen, the TPC of the *P. amaryllifolius* leaves extract increased sharply when raising the ethanol concentration from 0 to about 70% and then kept stably at the higher ratio of the alcohol. Meanwhile, the rise in temperature from 30°C to 60°C might enhance the TPC of the extract, while a higher temperature might decrease the phenolic contents due to the decomposition of some metabolites. Besides, the temperature approximated the boiling point of ethanol led to a decrease in the extraction yield due to the fast evaporation of the solvent and a longer extraction time also affected certainly the TPC values of the extract. From the numerical optimization, the maximum predicted TPC of the phenolic enrichment at 93.59 mgGAE/g could be obtained under the conditions of 61.18°C extraction temperature, 163.00-min extraction time, and 71.79% ethanol concentration. The actual condition was slightly modified at 65°C, ethanol 70%, and an extraction time of 160 min. The TPC of the phenolic enrichment under the optimal condition was 105.69 mgGAE/g, which was higher than the calculated value above.

### 3.3. Chemical Composition of the Phenolic Enrichment From the *P. amaryllifolius* Leaves

The phenolic enrichment was further isolated for the determination of its chemical composition. The alkaloid fraction was analyzed in comparison with the reference compounds as reported in [15] using the HPLC-DAD method. The analytical data identified pandalizine A, pandalizine B, pandamarilactione B, pandamarilactone-1, pandamarilactione G, and dubiusamine A in the alkaloids fraction. This result indicated that a certain amount of alkaloids still presented in the contents and may affect to the bioactivities of the phenolic enrichment from the *P. amaryllifolius* leaves.

**Figure 3:**
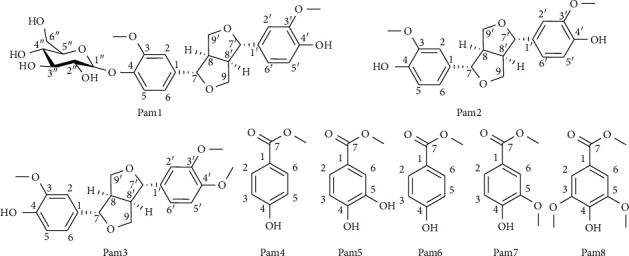
Structures of isolated compounds from the phenolic enrichments of *P. amaryllifolius* leaves.

Meanwhile, from the nonalkaloid fraction, eight phenolics were isolated and their structures (Figure 3) were elucidated using spectroscopy methods.

Compound **Pam1** was obtained as a white powder. The molecular weight of **Pam1** was 520 Da by the ESI-MS spectrum (Figure S1) at *m/z* 521 [M + H]^+^ and *m/z* 1041 [2M + H]^+^. The ^1^H-NMR spectrum (Figure S2) indicated two ABX systems at δ_H_ 7.05 (1H, d, *J = *2.0 Hz, H- 2), 7.17 (1H, d, *J = *8.5 Hz, H-5), 6.94 (2H, dd, *J = *8.5; 2.0 Hz, H-6, H-6′), 6.97 (1H, d, *J = *2.0 Hz, H-2′), 6.79 (1H, d, *J = *8.5 Hz, H-5′); a sugar unit identified as *β-D-*glucopyranoside based on the anomeric proton signals at δ_H_ 4.91 (1H, d, *J = *7.5 Hz, H-1″) and other signals ranging from 3.30 ppm to 3.46 ppm; two methoxy groups δ_H_ 3.86 (3H, s, 3′-OMe) and 3.84 (3H, s, 3-OMe); and two oxygenated methine groups δ_H_ 4.73 (1H, d, *J = *4.5 Hz, H-7′), 4.78 (1H, d, *J = *4.5 Hz, H-7). The ^13^C-NMR and DEPT spectra showed 26 carbon signals of which 12 carbon signals at δ_C_ 111.6–151.0 (6 CH, 6 C) confirmed the presence of two ABX systems. In addition to the typical signals of *β*-D-glucopyranoside at δ_C_ 102.9 (C-1″); 77.9 (C-3″); 74.9 (C-2″); 71.4 (C-4″); 78.2 (C-5″); 62.5 (C-6″), in the ^13^C NMR spectrum (Figure S3), there were signals of two methine groups, two methylene groups attached to oxygen, respectively, at δ_C_ 87.4 (C-7); 87.1 (C-7′); 72.7 (C-9, C-9′), and two methoxy groups at δ_C_ 56.8 (3′-OMe); 56.4 (3-OMe). The structure of **Pam1** was determined to be pinoresinol 4*-O-β-D*-glucopyranoside by comparison of spectral data with those reported in the literature [20].

Compound **Pam2** was obtained as a light yellow oil. The ^1^H-NMR spectrum of **Pam2** (Figure S5) exhibited signals of an ABX system due to three protons *δ*_H_ 7.00 (2H, s, H-2, 2ʹ); 6.82 (2H, d, *J = *8.5 Hz, H-6, 6ʹ), and *δ*_H_ 6.89 (2H, d, *J = *8.0 Hz, H-5, 5′), an oxymethine group *δ*_H_ 4.73 (2H, d, *J = *4.0 Hz, H-7, 7ʹ). Additionally, the spectrum showed two oxymethylene signals *δ*_H_ 3.88 (2H, dd, *J = *9.0; 4.0 Hz, H-9a, 9′a) and 4.26 (2H, dd, *J = *9.0; 6.5 Hz, H-9b, 9′b), a methine group *δ*_H_ 3.05 (2H, m, H-8, 8ʹ), and two methoxy groups at *δ*_H_ 3.91 (3H, s) and 3.89 (3H, s). Besides, the ^13^C-NMR and DEPT spectrum (Figure S6) showed 10 carbons, including three aromatic -CH groups *δ*_C_ 108.8 (C-2, 2′), 114.4 (C-5, 5′), and 119.4 (C-6, 6′), three nonprotonated carbons 133.1 (C-1, 1′), two aromatic rings directly associated with oxygen *δ*_C_ 145.4 (C-3) and 146.9 (C-4), and two methoxy groups at *δ*_C_ 56.1 Two methine signals *δ*_C_ 54.3 (C-8, 8′), an oxymethine group at *δ*_C_ 86.0 (C-7, 7′), and an oxymethylene group *δ*_C_ 71.6 (C-9, C-9ʹ) suggested a structure of a benzofuran derivative. In addition, the molecular weight of **Pam2** was determined as 358 Da corresponding with the formula C_20_H_22_O_6_ based on the ions at *m/z* 359 [M + H]^+^, *m/z* 739 [2M + Na]^+^, *m/z* 341 [M-H_2_O + H]^+^on the ESI-MS spectrum (Figure S4). The number of carbon and hydrogen in the predicted formula was twice as much as the number shown in the spectrum of **Pam2**; hence, it is possibly indicated that this compound has an axially symmetric benzofuran structure and the signals in the NMR spectrum were double shorted. The spectral data of **Pam2** resembled the data of pinoresinol in previous publications [20], so it can be identified as pinoresinol.

Compound **Pam3** was isolated as a yellowish-brown oil. The molecular weight of **Pam3** was 372 Da suggesting the formula was C_21_H_24_O_6_ as determined from the ESI-MS spectrum (Figure S7) at *m/z* 373 [M + H]^+^, and *m/z* 395 [M + Na]^+^. Six proton signals in the ^1^H-NMR spectrum (Figure S8) *δ*_H_ 6.91 (1H, s, H-2′), 6.90 (1H, s, H-2), 6.89 (1H, d, *J = *9.0 Hz, H-5), 6.85 (1H, d, *J = *9.0 Hz, H-5′), 6.87 (1H, dd, *J = *9.0; 2.0 Hz, H-6′), and 6.83 (1H, dd, *J = *9.0; 2.0 Hz) suggested an ABX system. Additionally, the ^1^H-NMR spectrum showed an oxymethine group *δ*_H_ 4.73 (2H, d, *J = *4.0 Hz, H-7, 7ʹ); two oxymethylene groups *δ*_H_ 4.25 (2H, m, H-9), and 3.87 (2H, m, H-9′); two methine groups *δ*_H_ 3.31 (2H, m, H-8, 8ʹ); and three methoxy groups at *δ*_H_ 3.91 (3H, s), 3.90 (3H, s), and 3.89 (3H, s). Besides, 21 carbon signals including six aromatic carbons *δ*_C_ 108.7 (C-2), 109.3 (C-2′), 114.3 (C-5), 111.1(C-5′), 119.0 (C-6), and 118.3 (C-6′); six nonprotonated aromatic carbons *δ*_C_ 133.6 (C-1), 132.9 (C-1′); four aromatic carbons directly bonding with oxygen *δ*_C_ 148.7 (C-3), 149.3 (C-3′), 145.3 (C-4), and 146.8 (C-4′); and three methoxy groups at *δ*_C_ 56.0 were observed in the ^13^C-NMR and DEPT spectrum (Figure S9). Two methine groups *δ*_C_ 54.2 (C- 8, 8′), two oxymethine groups *δ*_C_ 85.8 and 85.9 (C-7, 7′), two oxymethylene groups *δ*_C_ 71.7 (C-9, C-9ʹ) allowed the identification of two benzofuran rings. It can be seen that half of the number of NMR and MS data of **Pam3** were similar to those of **Pam2**, except for an additional methoxy group. Based on spectral analysis and comparison with the literature [21], the structure of **Pam3** was determined to be pinoresinol monomethyl ether.

The rest of the compounds, including methyl 4-hydroxybenzoate **(Pam4)**, 3,4-dihydroxyl benzoate methyl **(Pam5)**, 4-hydroxy benzoic acid **(Pam6)**, methyl 4-hydroxy-3-methoxybenzoate **(Pam7)**, and methyl syringate **(Pam8)** were identified by comparing their NMR (Figure S10–S24) data to those of reported studies [22–26]. Previously, flavonoids, coumaroyl malate, coumarin derivatives, and some other phenolics have been investigated from *P. amaryllifolius* leaves [2, 8, 9]. In the current study, the phytochemical investigation of the phenolic enrichment from *P. amaryllifolius* leaves led to the isolation of 8 compounds, including 3 lignans and 5 benzoate derivatives, whereas, except for 4-hydroxy benzoic acid **(Pam6)**, the other phenolics were identified for the first time from the plant.

### 3.4. Antioxidant and NO Production Inhibition of the Phenolic Enrichment

The isolated lignans, such as pinoresinol 4*-O-β-D*-glucopyranoside (**Pam1**), pinoresinol (**Pam2**), and pinoresinol monomethyl ether (**Pam3**) have been proven as significant antioxidant compounds [27–29], while other *p*-hydroxybenzoic derivatives also had considerable free-radical scavenging effects [30, 31]. In this study, the phenolic enrichment of *P. amaryllifolius* leaves, which contained these compounds, was evaluated for its antioxidant activity via DPPH and hydroxy free radical scavenging effects (Table 3). Besides, the NO production inhibition of the phenolic enrichment was evaluated and compared with those of the alkaloid and nonalkaloid fractions.

The phenolic enrichment from *P. amaryllifolius* leaves exhibited significant antioxidant and NO inhibition activities compared to both its derived alkaloid and nonalkaloid fractions as well as the positive controls used in this study. For DPPH and hydroxyl radical scavenging assays, the phenolic enrichment displayed IC_50_ values of 32.68 and 40.20 μg/mL, respectively, which are close to the efficacy of ascorbic acid (IC_50_ of 24.65 μg/mL) and catechin (IC_50_ of 32.27 μg/mL), respectively. This suggests that the phenolic enrichment possesses potent free radical scavenging abilities, on par with well-known antioxidants. In terms of NO inhibition, the phenolic enrichment achieved an IC_50_ of 21.44 μg/mL, which, while not as potent as cardamonin (IC_50_ of 2.98 μg/mL), is markedly more effective than either the nonalkaloid fraction (57.61 μg/mL) or the alkaloid fraction (33.47 μg/mL). This indicates that while cardamonin serves as an exceptionally potent NO inhibitor, the phenolic enrichment's moderate yet significant inhibition points to a synergistic effect within the mixture, potentially due to the interplay between phenolic and alkaloid components.

The higher NO inhibition in the phenolic enrichment compared to its separated fractions suggests a synergistic interaction between phenolic and alkaloid compounds within the mixture, enhancing the NO inhibition capacity beyond what is observed in either fraction alone. Although individual compounds like cardamonin show stronger inhibition, the phenolic enrichment still represents a balanced and effective option for reducing oxidative stress and inflammation, leveraging the combined effects of its components. This highlights the potential of using phenolic enrichment as a natural source of antioxidants and anti-inflammatory agents, with a broader range of bioactivity than isolated components.

## 4. Conclusion

The optimized phenolic enrichment from *P. amaryllifolius* leaves demonstrated substantial antioxidant and NO inhibition activities, surpassing the efficacy of its individual nonalkaloid and alkaloid fractions. The phenolic enrichment enhanced NO inhibition capacity suggests a synergistic interaction between its phenolic and alkaloid compounds, contributing to a broader range of bioactivity. While the enrichment exhibited significant antioxidant effects comparable to standard antioxidants like ascorbic acid and catechin, its NO inhibition effect, though less than cardamonin, was markedly superior to that of the isolated fractions. These findings support the use of phenolic enrichment as a balanced and effective option for managing oxidative stress and inflammation, highlighting its potential as a multifunctional natural therapeutic agent.

## Figures and Tables

**Table 1:** Levels of the variables in Box–Behnken design.

Variables	Unit	Code levels		
		−1	0	1
Temperature (*X*_1_)	°C	30	55	80
Ethanol concentration (*X*_2_)	%	0	48	96
Time (*X*_3_)	minutes	60	180	300

**Table 2:** Responses of TPC of the extracts to independent variables using Box–Behnken design.

No.	Variables			TPC (mg GAE/g)	
	*X*_1_: Temp. (°C)	*X*_2_: EtOH (%)	*X*_3_: Time (min)	Experimental value	Predicted value
1	55	48	180	92.66	91.10
2	55	48	180	89.57	91.10
3	30	48	300	81.15	81.66
4	30	0	180	54.97	54.92
5	55	96	60	89.43	90.09
6	55	48	180	92.9	91.10
7	55	48	180	92.24	91.10
8	80	96	180	85.71	86.05
9	30	96	180	82.74	82.90
10	55	0	300	76.74	76.27
11	55	48	180	90.51	91.10
12	80	48	60	90.7	90.28
13	55	96	300	89.95	89.59
14	55	48	180	89.93	91.10
15	55	0	60	70.21	70.76
16	55	48	180	89.55	91.10
17	30	48	60	70.57	70.10
18	80	48	300	83.17	83.73
19	80	0	180	81.43	81.38

**Table 3:** Free radical scavenging effects of phenolic enrichment from *P. amaryllifolius* leaves.

Sample	DPPH (IC_50_, μg/mL)	Hydroxyl (IC_50_, μg/mL)	NO inhibition (IC_50_, μg/mL)
The phenolic enrichment	32.68 ± 2.93^a^	40.20 ± 3.84^e^	21.44 ± 2.61^h^
The nonalkaloid fraction	28.75 ± 1.81^b^	41.11 ± 3.76^e^	57.61 ± 4.54^i^
The alkaloid fraction	52.10 ± 4.26^c^	82.35 ± 6.09^f^	33.47 ± 2.92^j^
Ascorbic acid^∗^	24.65 ± 2.27^d^	—	
Catechin^∗∗^	—	32.27 ± 1.56^g^	
Cardamonin^#^			2.98 ± 0.35^k^

^∗,∗∗,#^Positive control.

^a–k^Data are expressed as mean ± SD. Means in each column with different letters are significantly different (*p* < 0.05).

## Data Availability

The data that support the findings of this study are available from the corresponding author upon reasonable request.

## Supporting Information

Detailed NMR data of the isolated compounds.
