# Supplementary material for: Optimization of the Extraction Conditions and Evaluation of Bioactivities of the Phenolic Enrichment From Pandanus amaryllifolius Leaves
Source: J Anal Methods Chem. 2025 May 7;2025:5256388. doi: 10.1155/jamc/5256388 (PMC12077974; doi:10.1155/jamc/5256388)
Supplement: Supporting Information — Additional supporting information can be found online in the Supporting Information section. [file 5256388.f1.docx]

**Supplemental**

**Optimization of the extraction conditions and evaluation of bioactivities of the phenolic enrichment from *Pandanus amaryllifolius* leaves.**

Do Hoang Giang^1,2^, Bui Thi Nhat Le^2^, Nguyen Thi Thu Minh^2^, Nguyen Thi Thu Thuy^3^, Nguyen Hai Dang^1^, Hoang Le Tuan Anh^2^, Nguyen Ngoc Tung^2^, Nguyen Tien Dat^2,*^

^1^ University of Science and Technology of Hanoi, Vietnam Academy of Science and Technology (VAST), 18-Hoang Quoc Viet, Cau Giay, Hanoi 10000, Vietnam

^2^ Center for High Technology Research and Development, VAST, 18-Hoang Quoc Viet, Cau Giay, Hanoi 10000, Vietnam.

^3^ Joint Vietnam-Russia Tropical Science and Technology Research Center, Nguyen Van Huyen, Cau Giay, Hanoi 10000, Vietnam

* Correspondence should be addressed to Nguyen Tien Dat, email: ngtiend@gmail.com

**Table of contents**

[Figure S1. The ESI-MS spectrum of **Pam1** 3](#_Toc188465642)

[Figure S2. The ^1^H NMR spectrum of **Pam1** 3](#_Toc188465643)

[Figure S3. The ^13^C NMR spectrum of **Pam1** 4](#_Toc188465644)

[Figure S4. The ESI-MS spectrum of **Pam2** 4](#_Toc188465645)

[Figure S5. The ^1^H NMR spectrum of **Pam2** 5](#_Toc188465646)

[Figure S6. The ^13^C NMR spectrum of **Pam2** 5](#_Toc188465647)

[Figure S7. The ESI-MS spectrum of **Pam3** 6](#_Toc188465648)

[Figure S8. The ^1^H NMR spectrum of **Pam3** 6](#_Toc188465649)

[Figure S9. The ^13^C NMR spectrum of **Pam3** 7](#_Toc188465650)

[Figure S10. The ESI-MS spectrum of **Pam4** 7](#_Toc188465651)

[Figure S11. The ^1^H NMR spectrum of **Pam4** 8](#_Toc188465652)

[Figure S12. The ^13^C NMR spectrum of **Pam4** 8](#_Toc188465653)

[Figure S13. The ESI-MS spectrum of **Pam5** 9](#_Toc188465654)

[Figure S14. The ^1^H NMR spectrum of **Pam5** 9](#_Toc188465655)

[Figure S15. The ^13^C NMR spectrum of **Pam5** 10](#_Toc188465656)

[Figure S16. The ESI-MS spectrum of **Pam6** 10](#_Toc188465657)

[Figure S17. The ^1^H NMR spectrum of **Pam6** 11](#_Toc188465658)

[Figure S18. The ^13^C NMR spectrum of **Pam6** 11](#_Toc188465659)

[Figure S19. The ESI-MS spectrum of **Pam7** 12](#_Toc188465660)

[Figure S20. The ^1^H NMR spectrum of **Pam7** 12](#_Toc188465661)

[Figure S21. The ^13^C NMR spectrum of **Pam7** 13](#_Toc188465662)

[Figure S22. The ESI-MS spectrum of **Pam8** 13](#_Toc188465663)

[Figure S23. The ^1^H NMR spectrum of **Pam8** 14](#_Toc188465664)

[Figure S24. The ^13^C NMR spectrum of **Pam8** 14](#_Toc188465665)

[Table S1. TPC of the extracts in the preliminary single-factor experiments 15](#_Toc188465666)


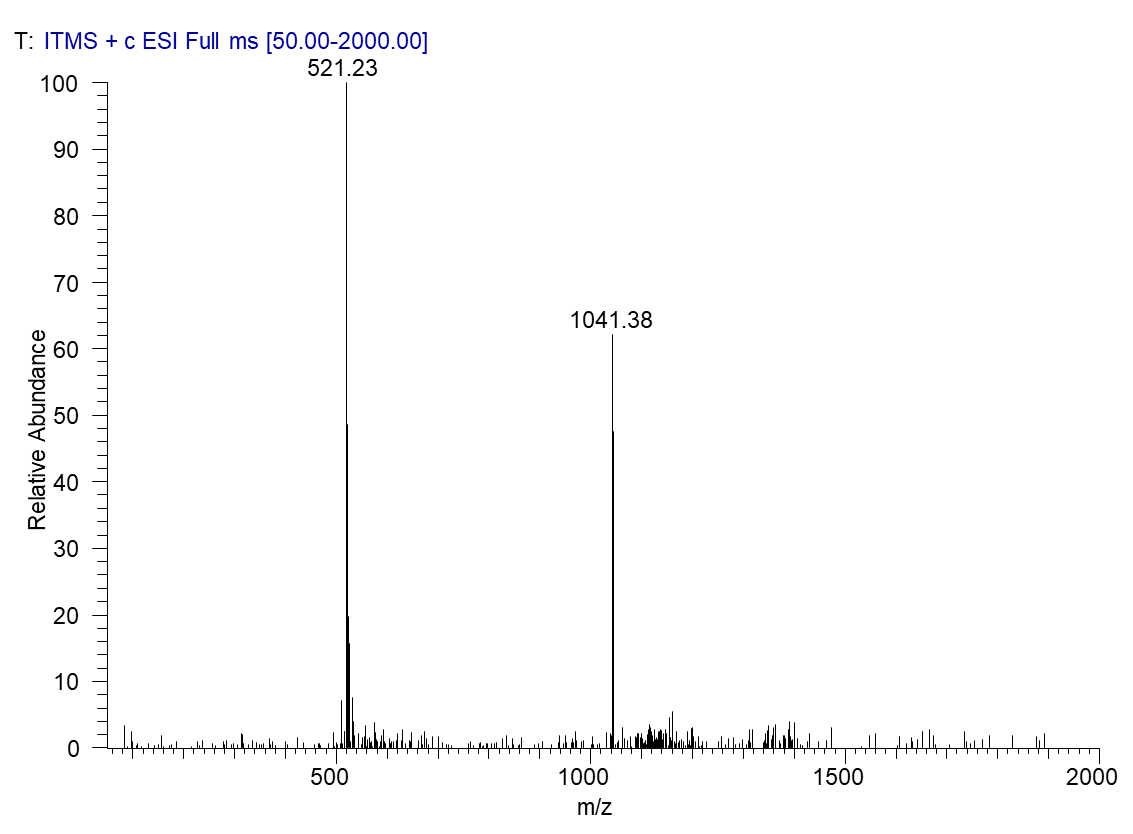


1. The ESI-MS spectrum of **Pam1**


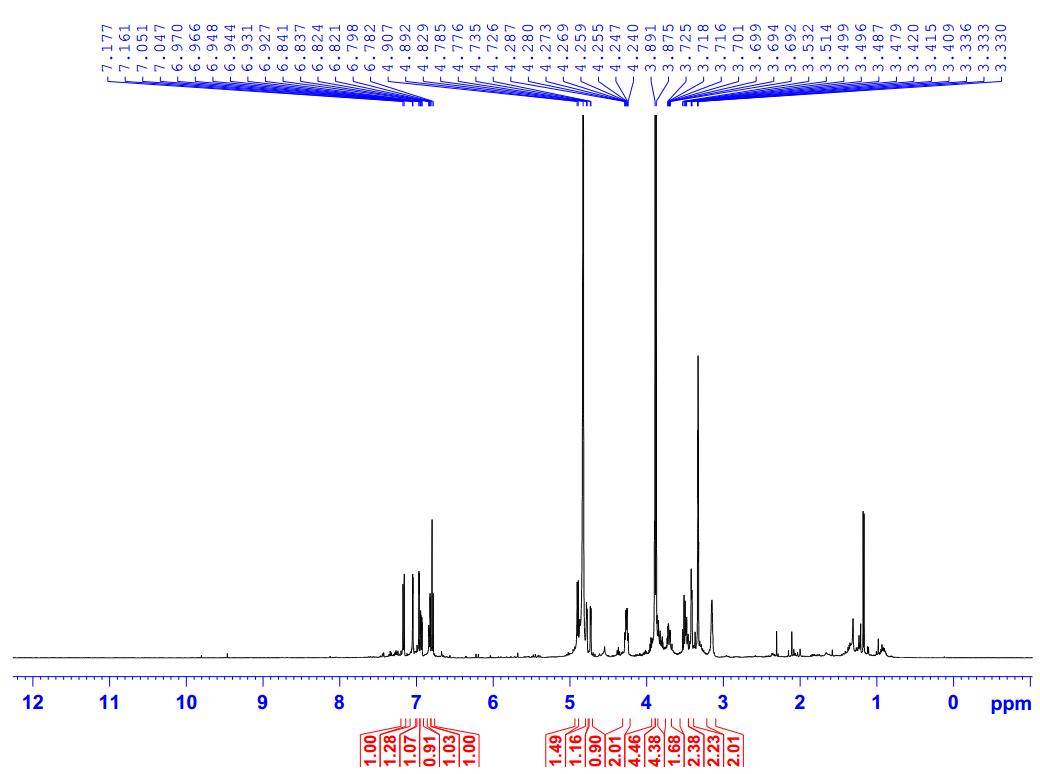


1. The ^1^H NMR spectrum of **Pam1**


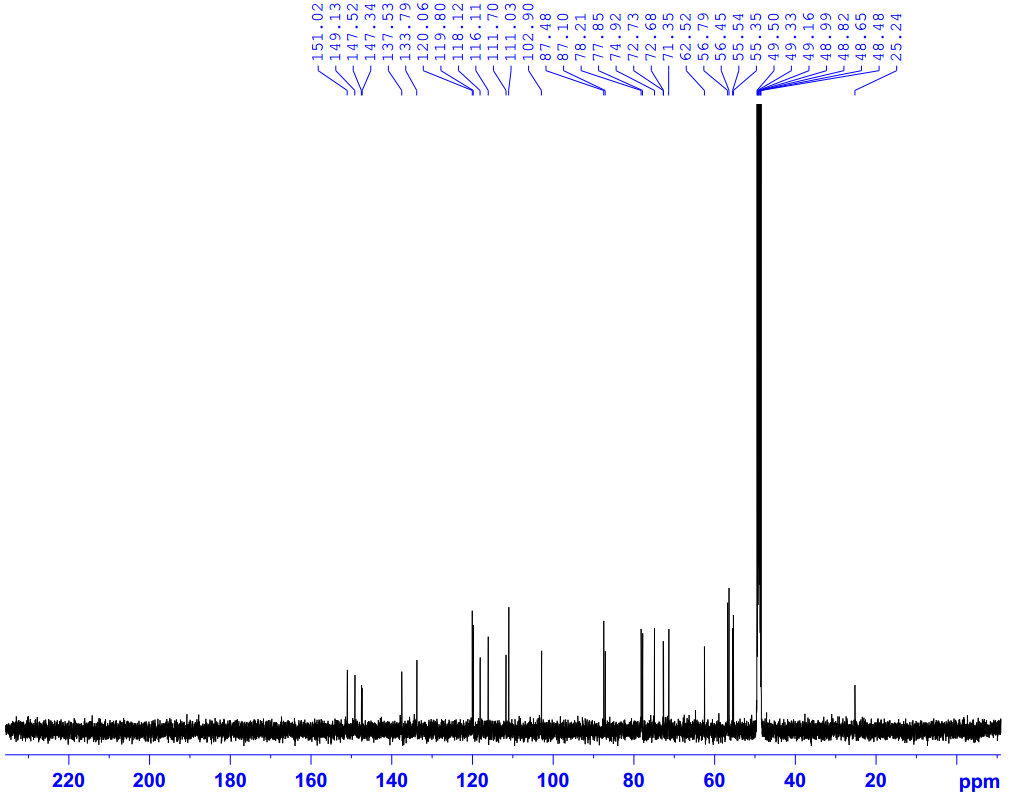


1. The ^13^C NMR spectrum of **Pam1**


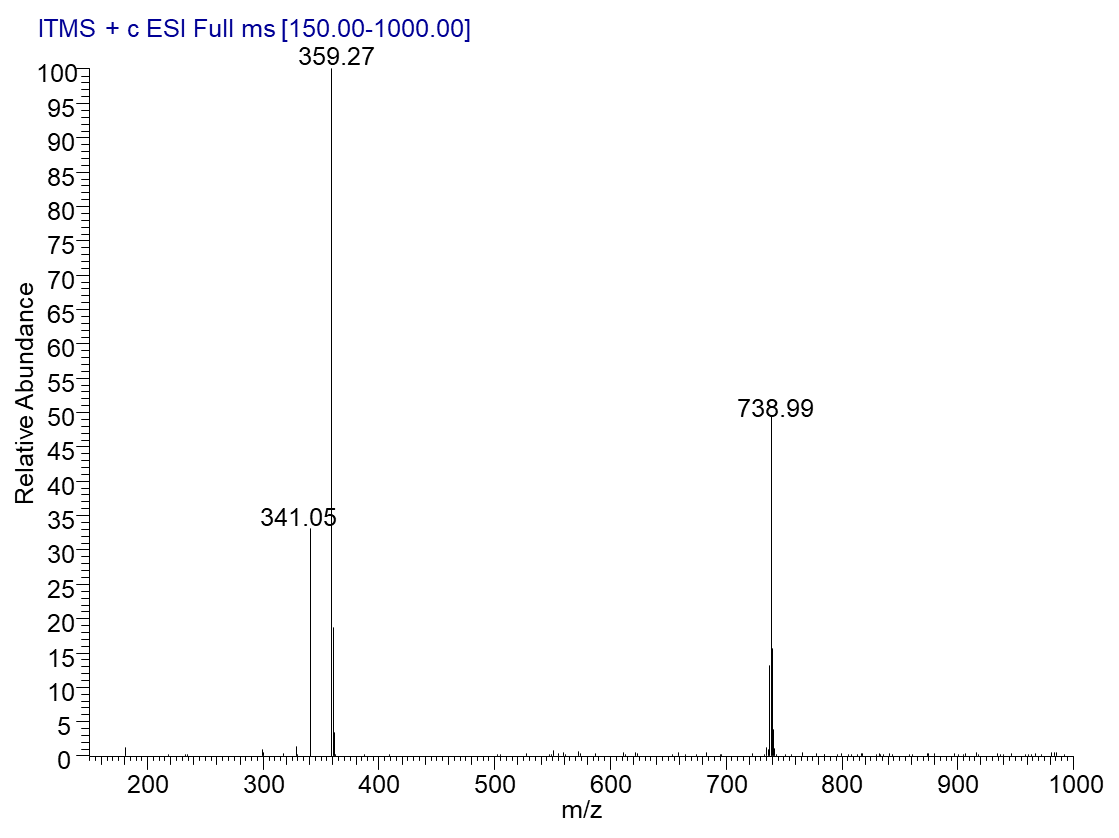


1. The ESI-MS spectrum of **Pam2**


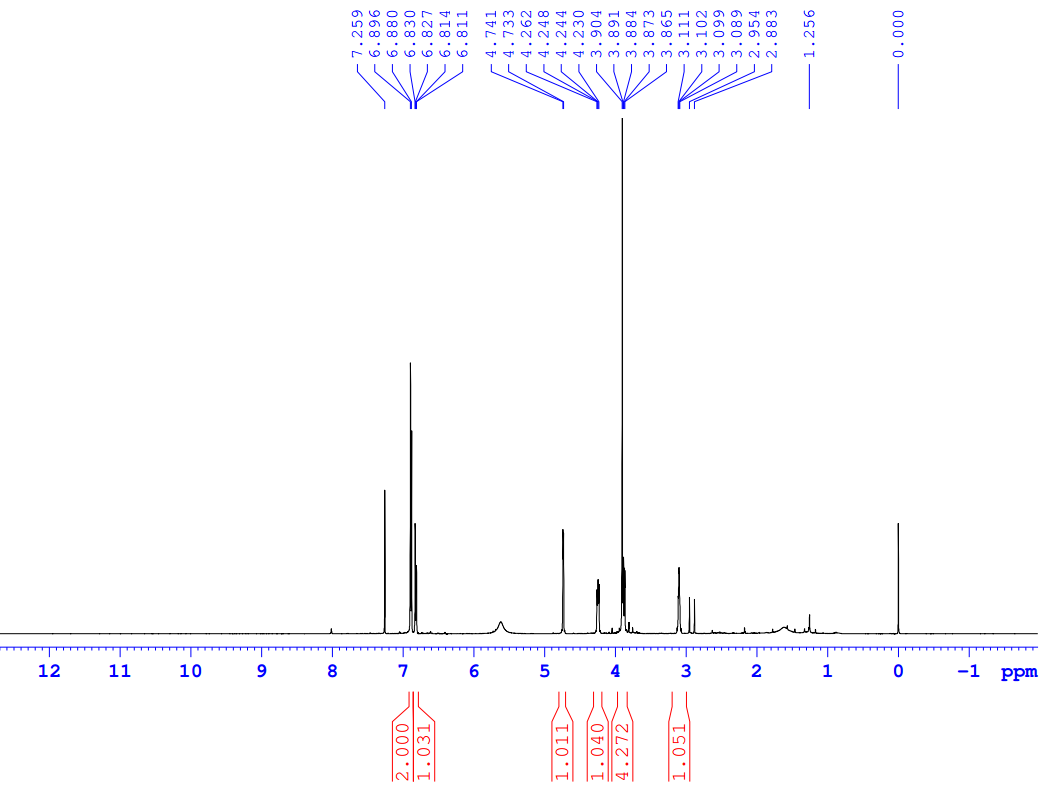


1. The ^1^H NMR spectrum of **Pam2**


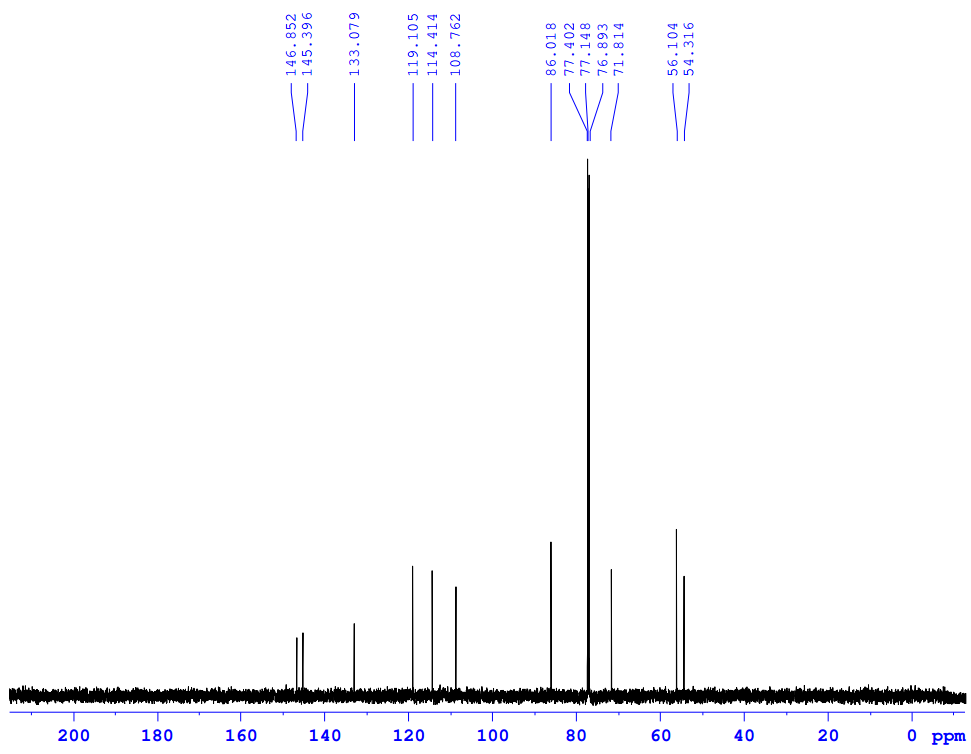


1. The ^13^C NMR spectrum of **Pam2**


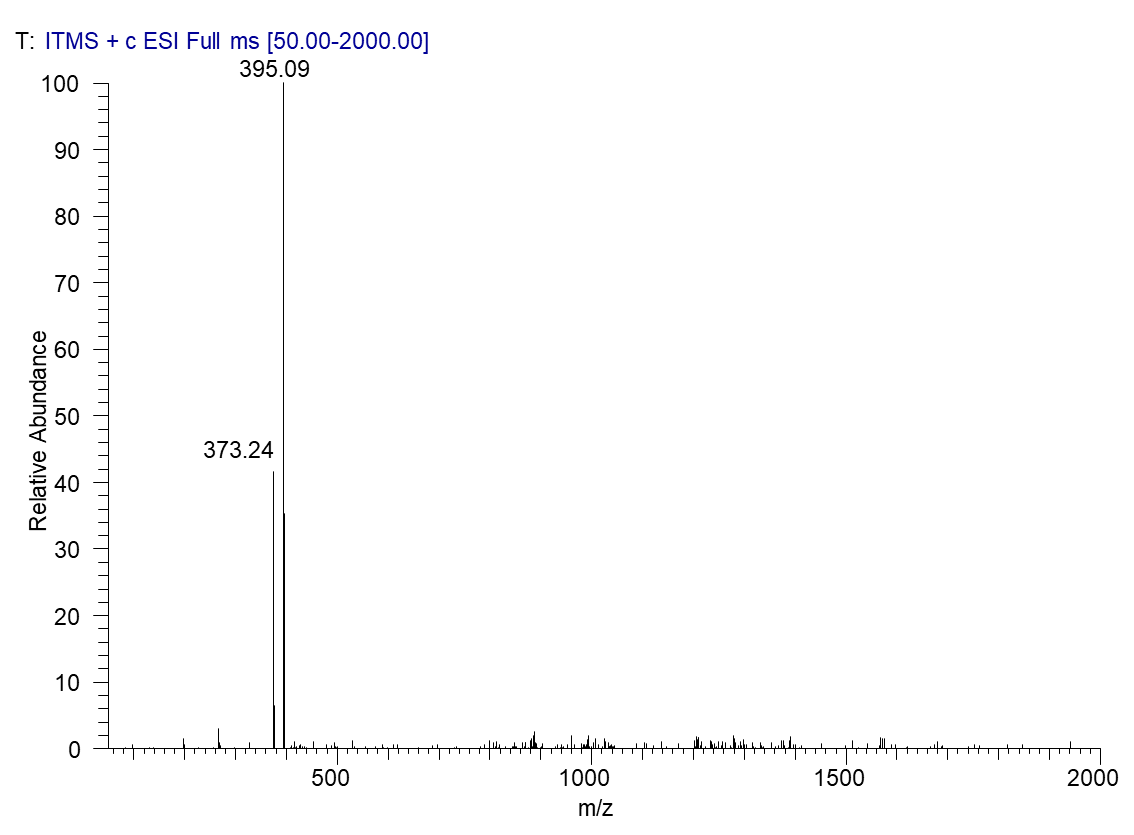


1. The ESI-MS spectrum of **Pam3**


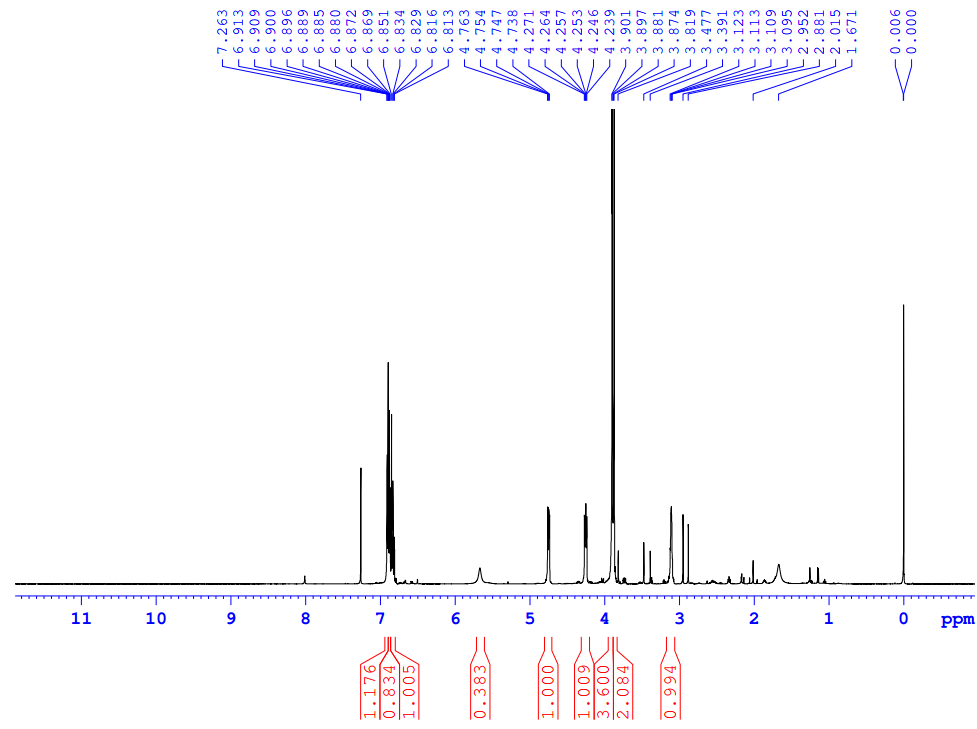


1. The ^1^H NMR spectrum of **Pam3**


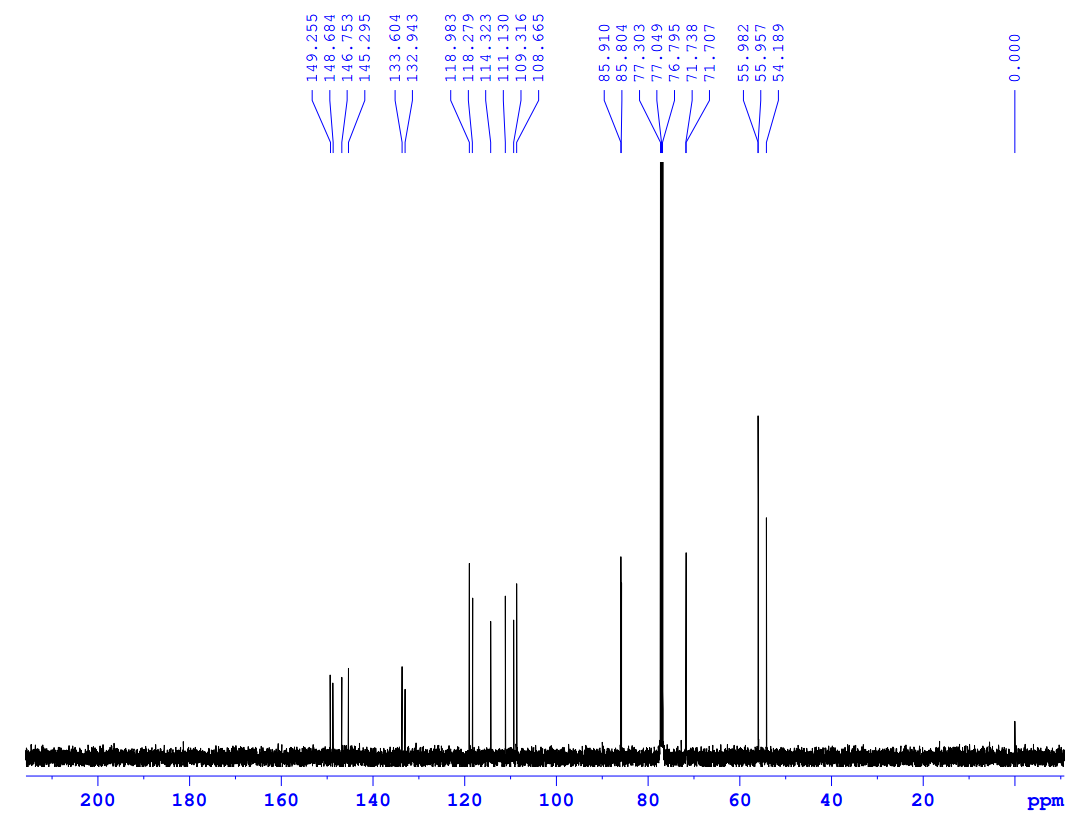


1. The ^13^C NMR spectrum of **Pam3**


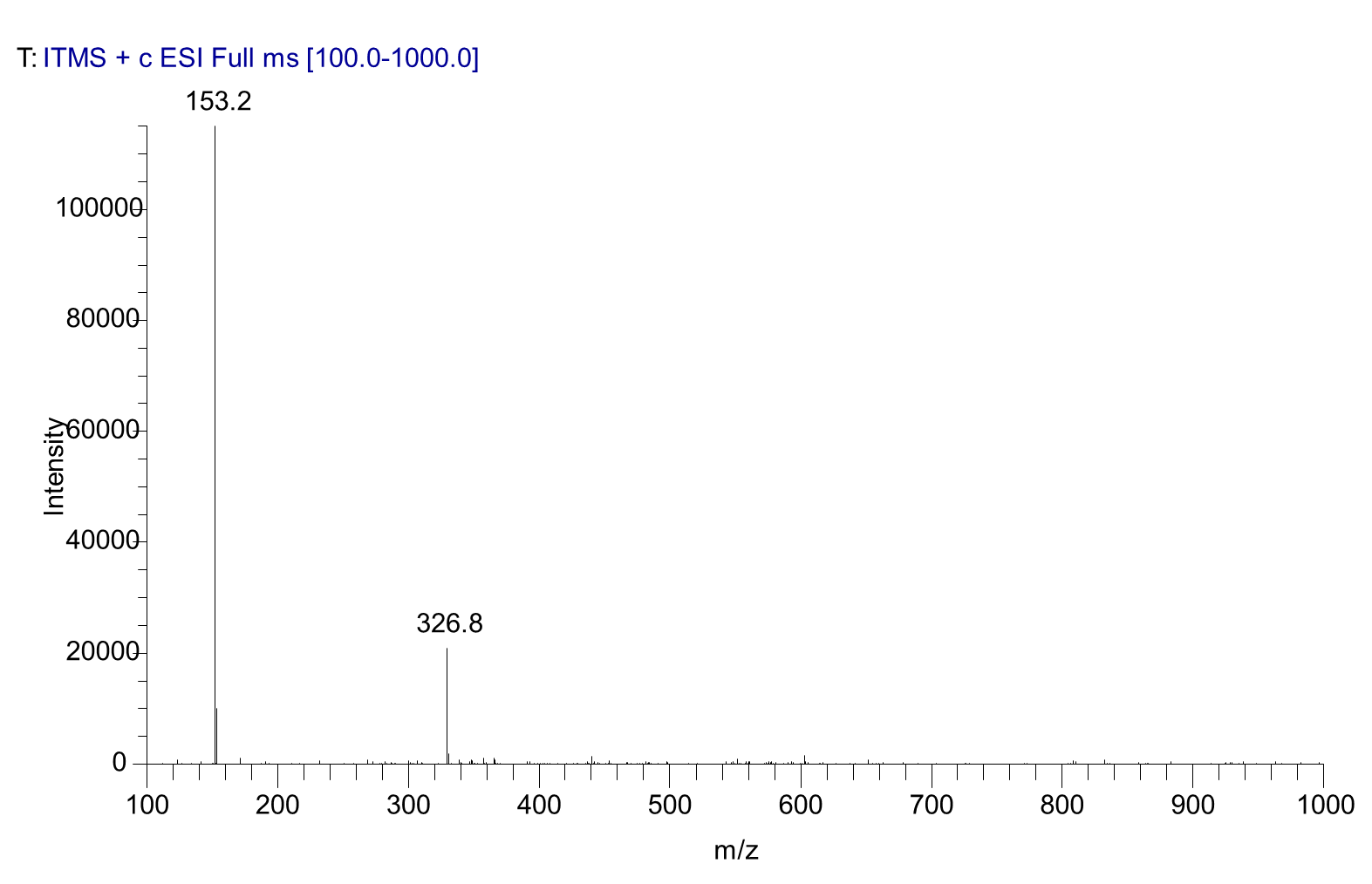


1. The ESI-MS spectrum of **Pam4**


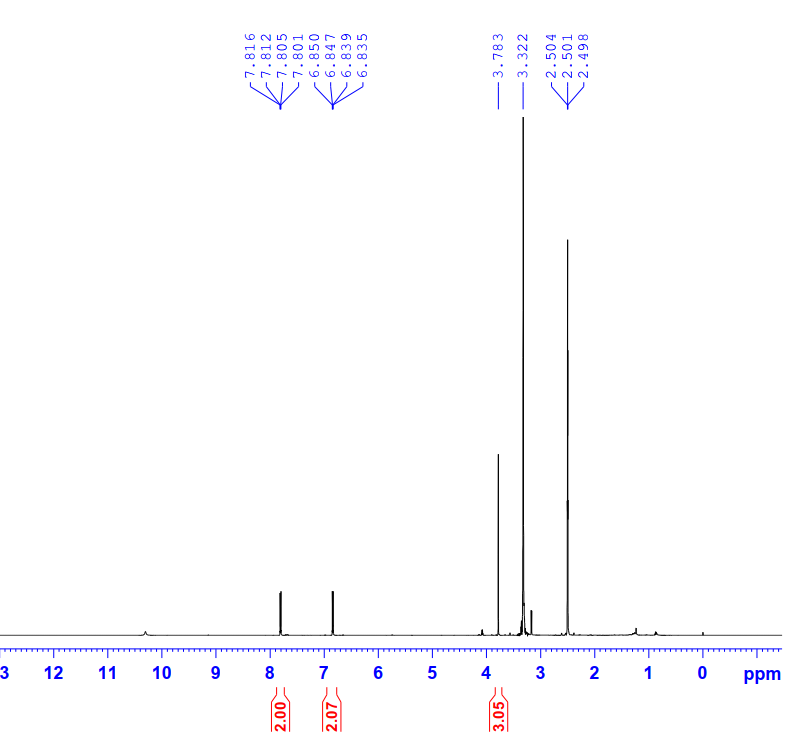


1. The ^1^H NMR spectrum of **Pam4**


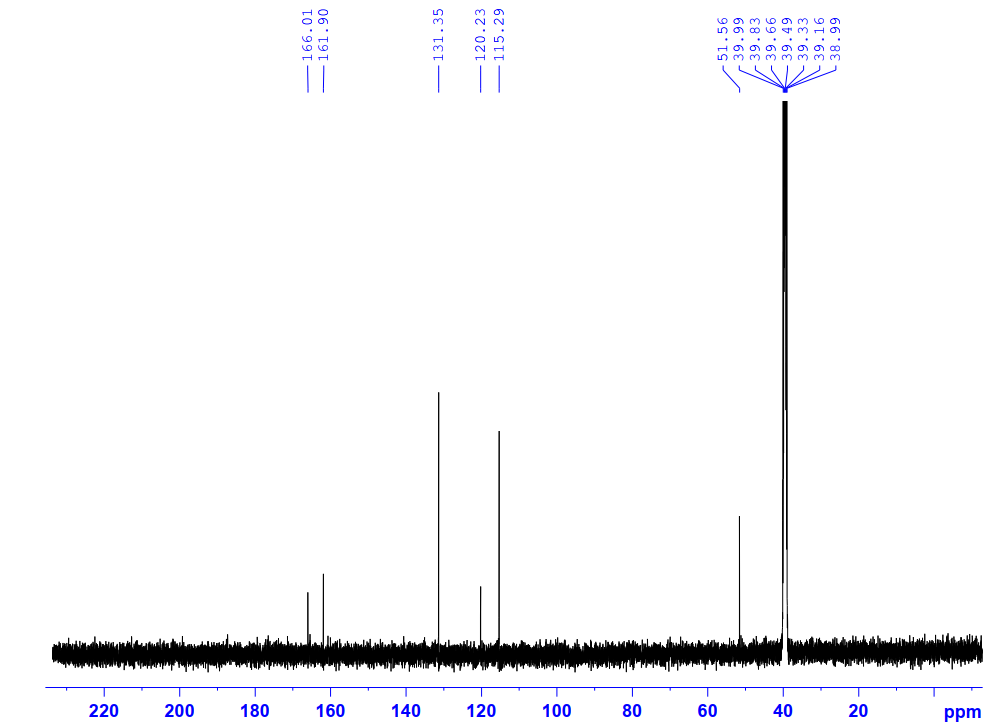


1. The ^13^C NMR spectrum of **Pam4**


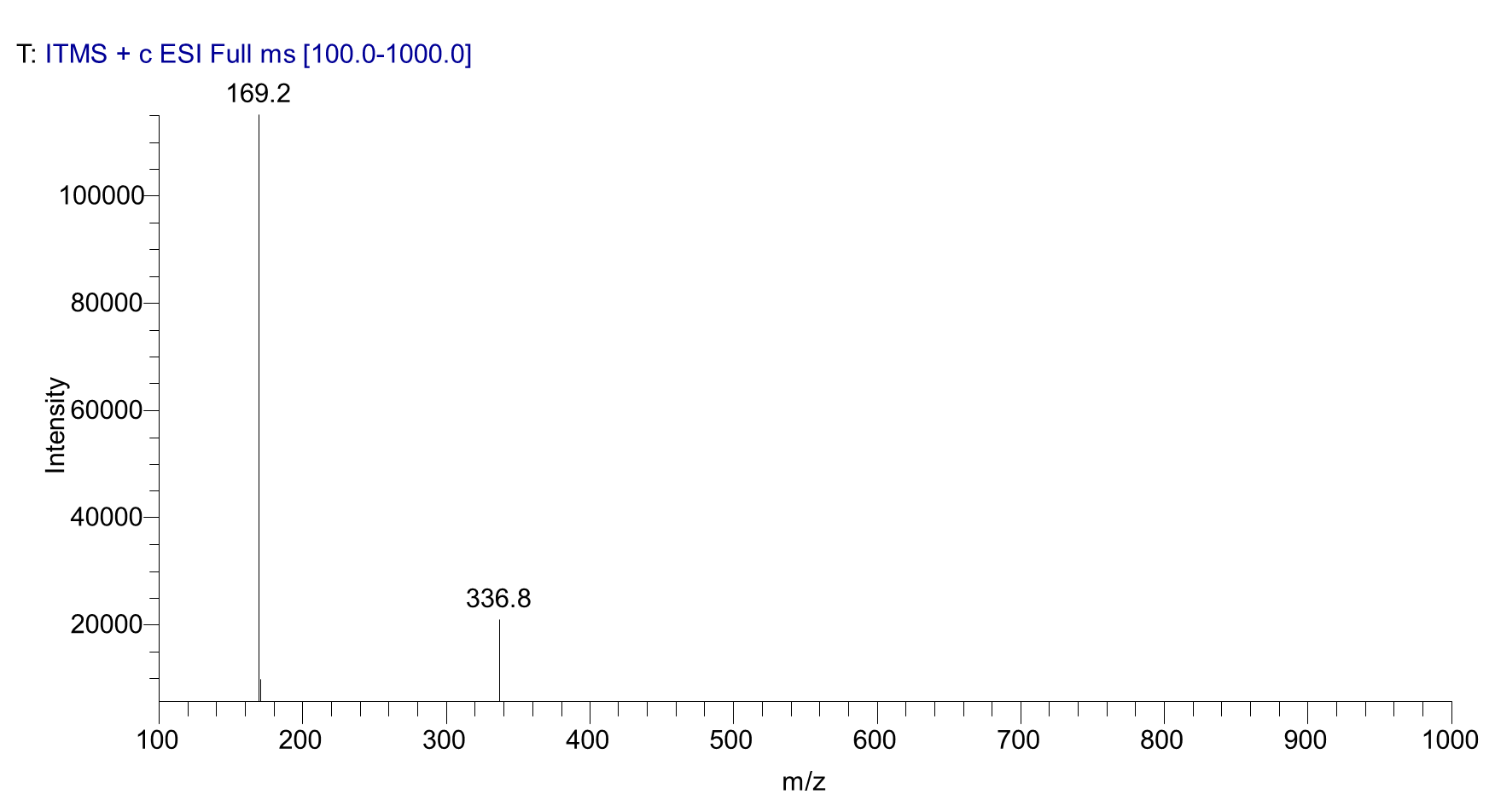


1. The ESI-MS spectrum of **Pam5**


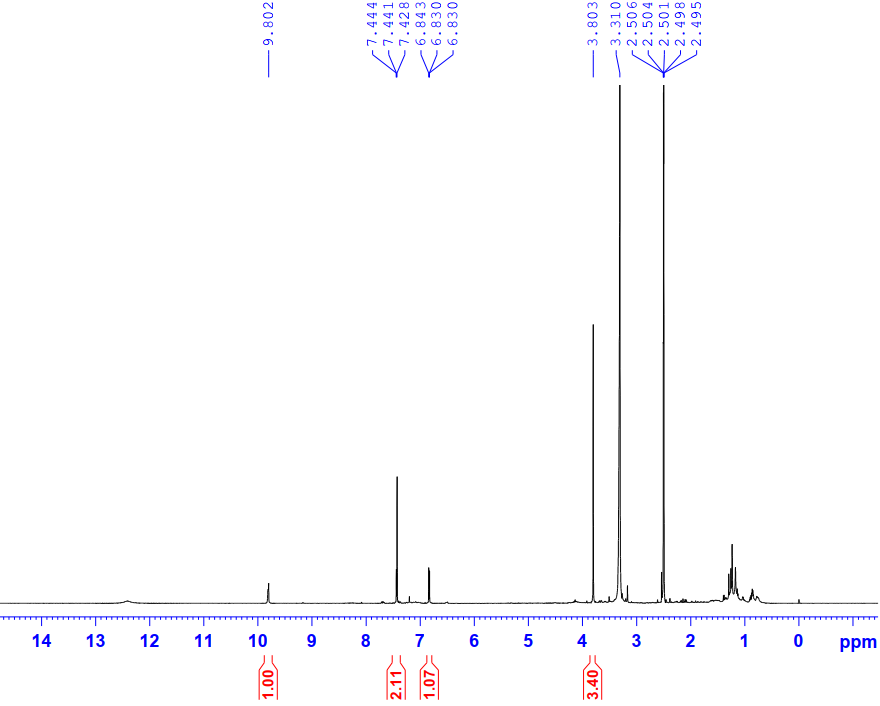


1. The ^1^H NMR spectrum of **Pam5**


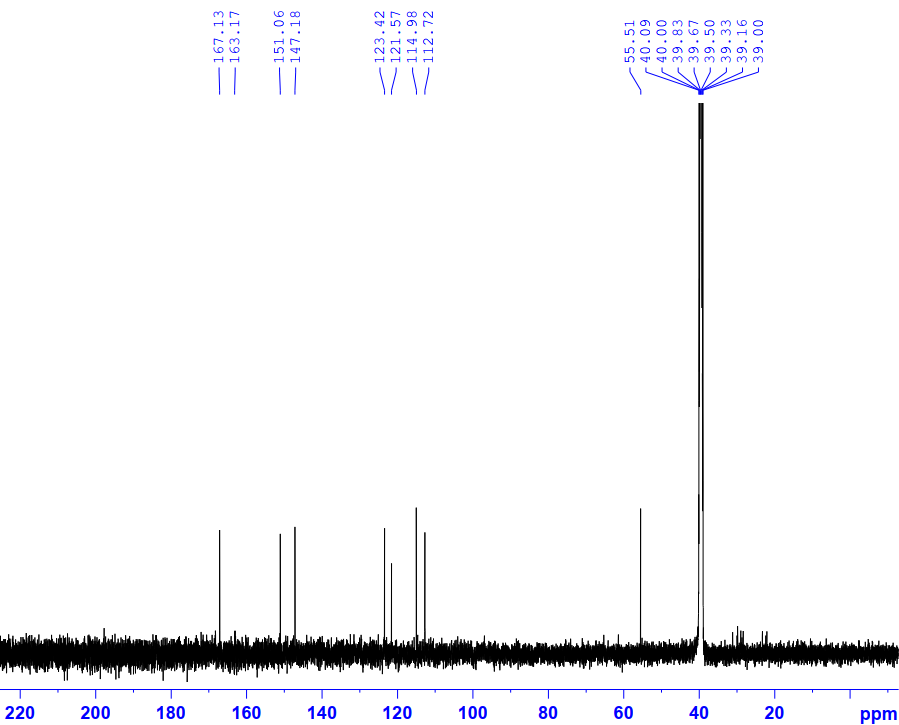


1. The ^13^C NMR spectrum of **Pam5**


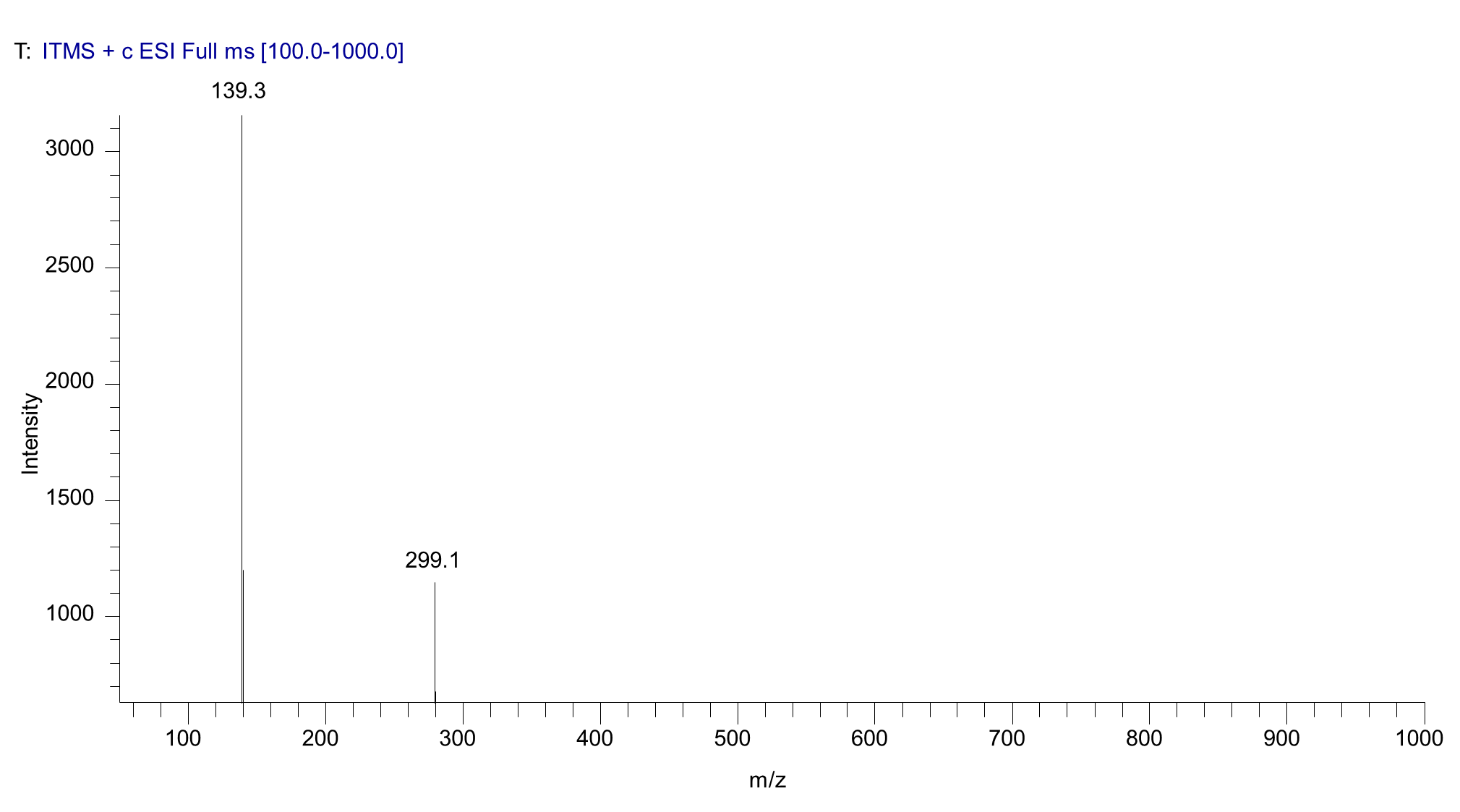


1. The ESI-MS spectrum of **Pam6**


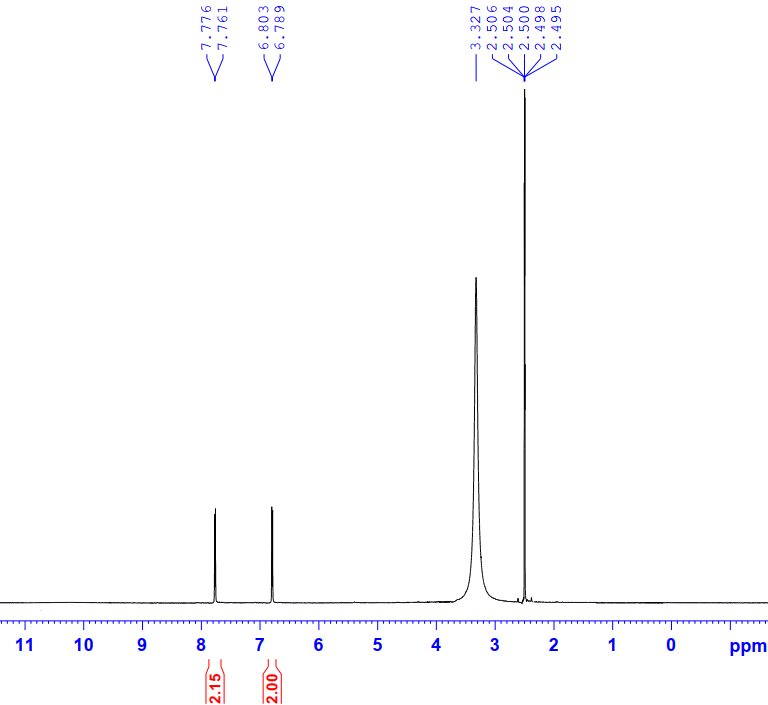


1. The ^1^H NMR spectrum of **Pam6**


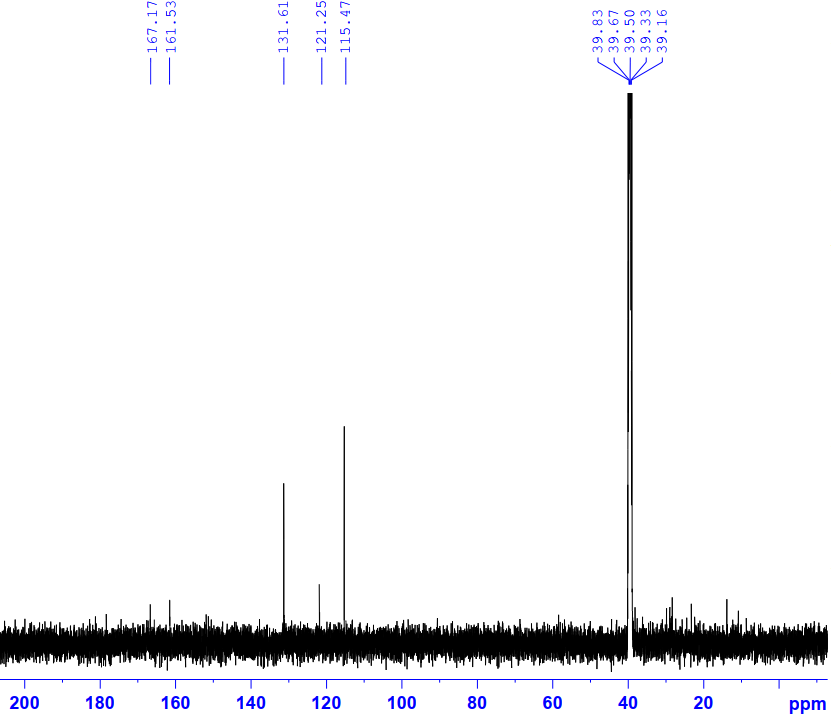


1. The ^13^C NMR spectrum of **Pam6**


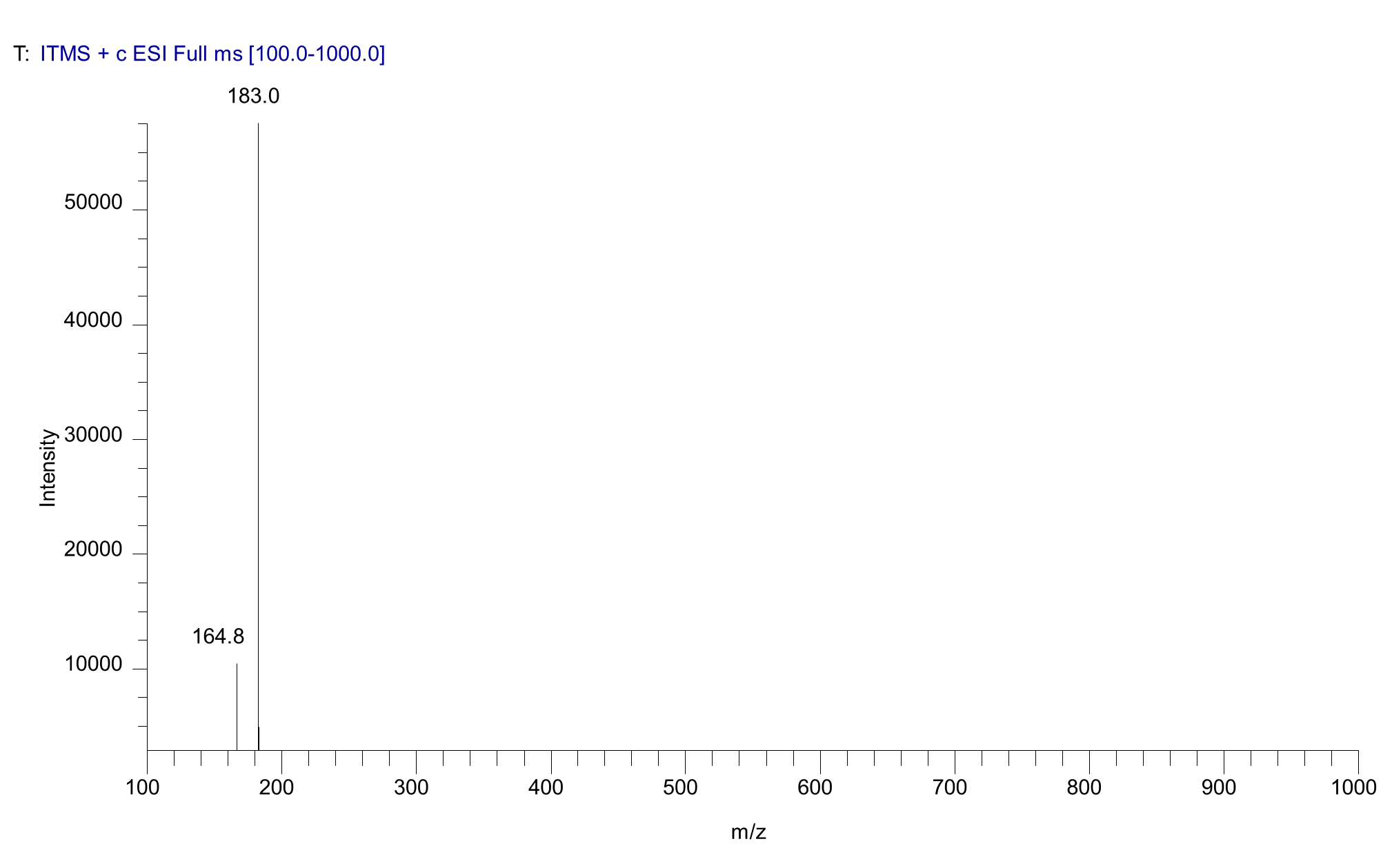


1. The ESI-MS spectrum of **Pam7**


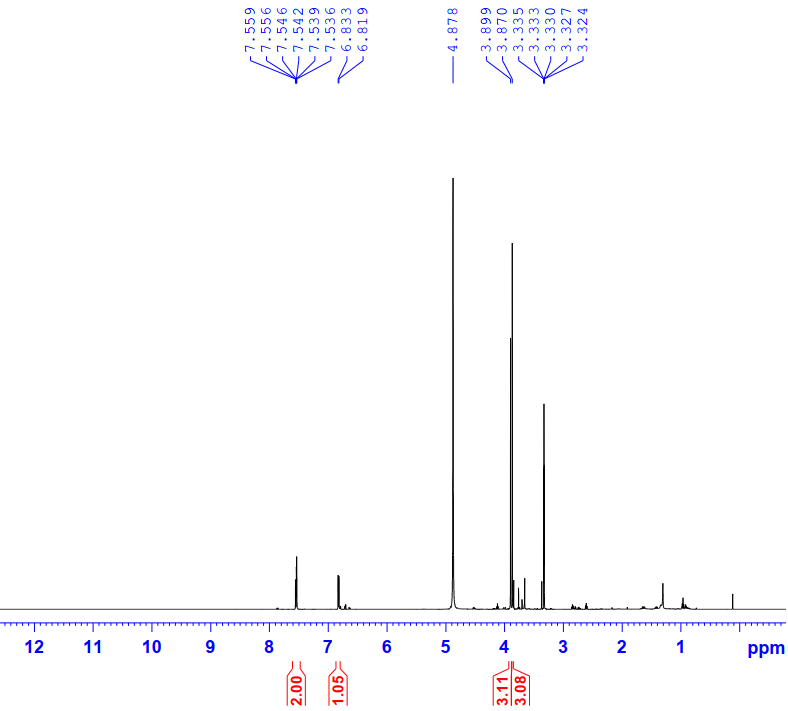


1. The ^1^H NMR spectrum of **Pam7**


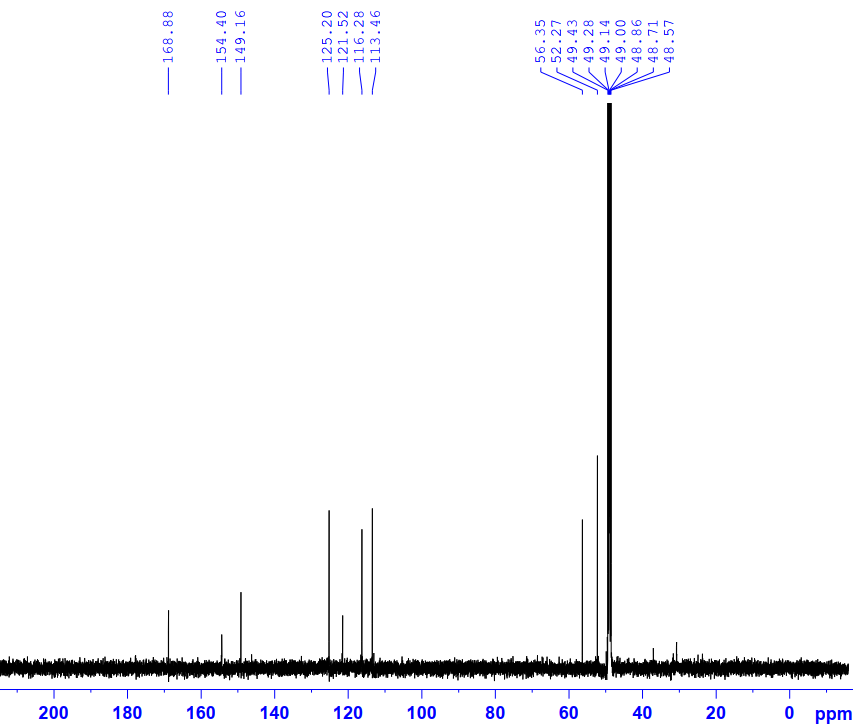


1. The ^13^C NMR spectrum of **Pam7**


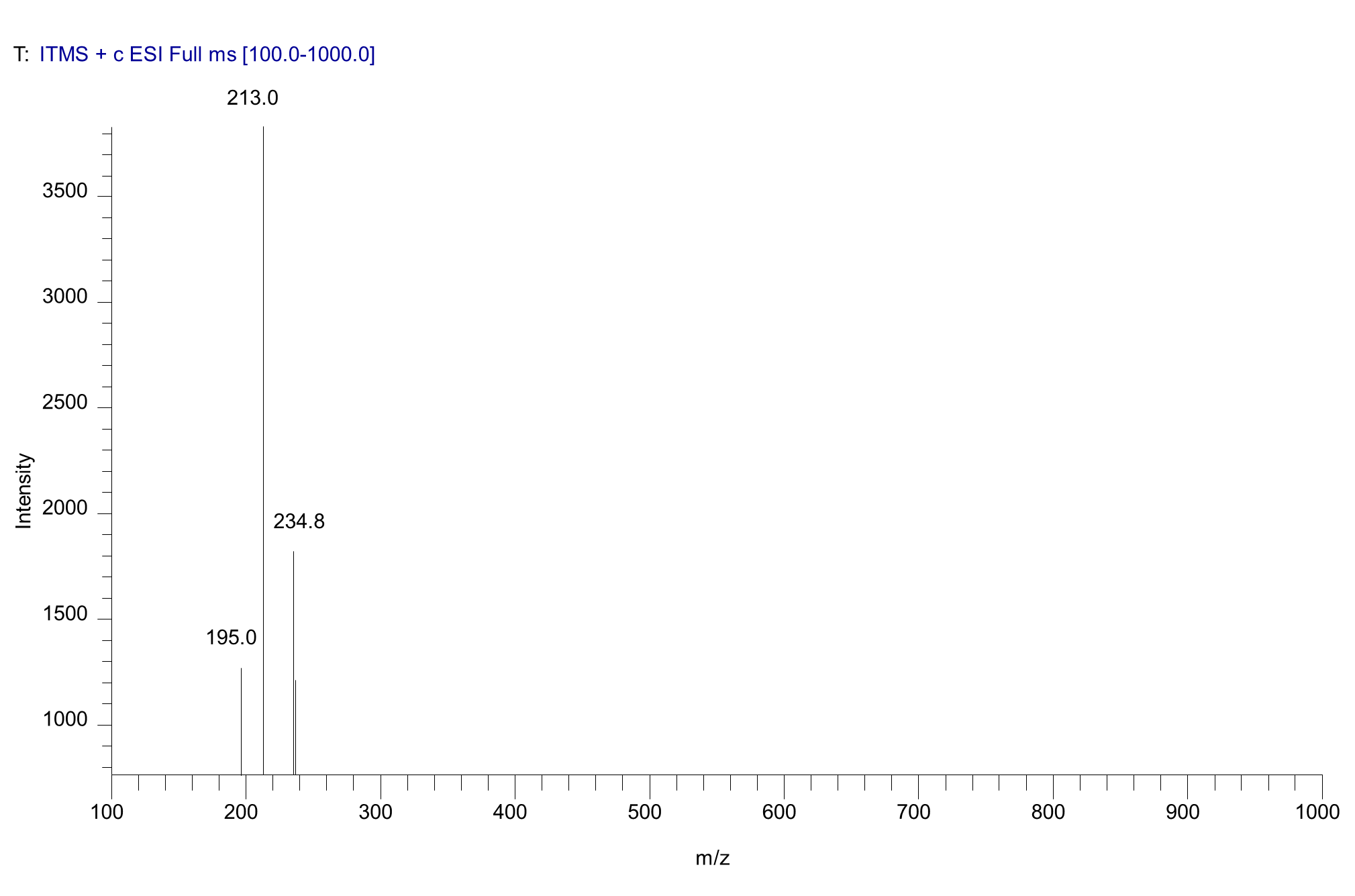


1. The ESI-MS spectrum of **Pam8**


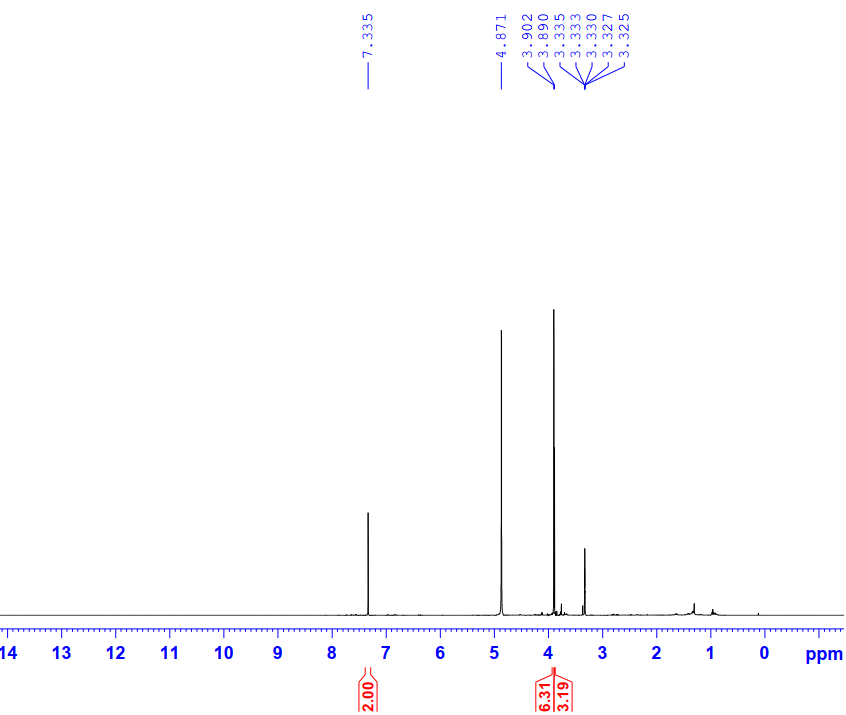


1. The ^1^H NMR spectrum of **Pam8**


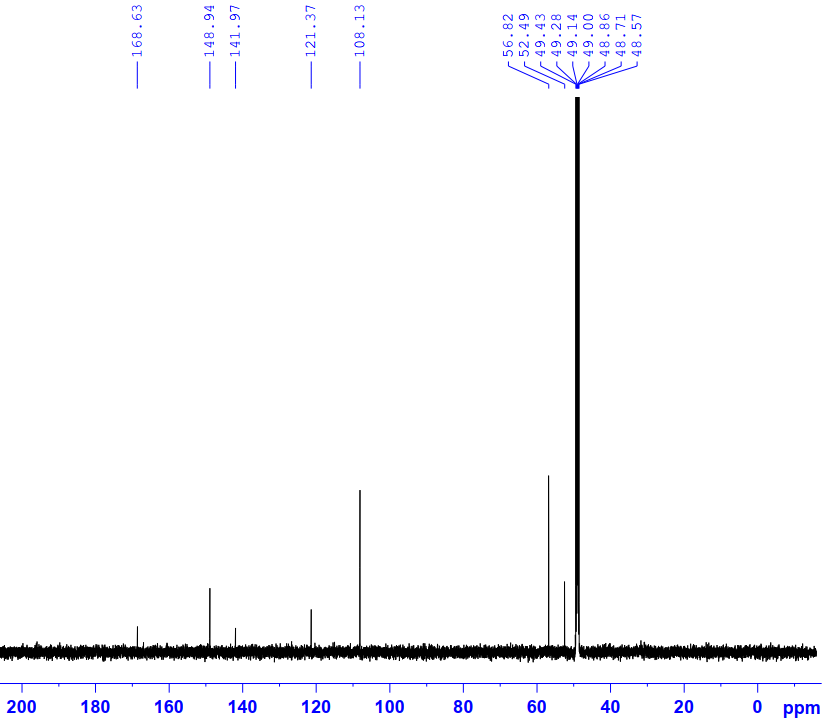


1. The ^13^C NMR spectrum of **Pam8**
2. TPC of the extracts in the preliminary single-factor experiments

| **Variables** | | | **TPC**  **(mg GAE/g)** |
| --- | --- | --- | --- |
| **X_1_: Temp. (°C)** | **X_2_: EtOH (%)** | **X_3_: Time (min)** |  |
| 30 | 96 | 180 | 85.04 |
| 40 | 96 | 180 | 87.47 |
| 50 | 96 | 180 | 90.46 |
| 60 | 96 | 180 | 92.22 |
| 70 | 96 | 180 | 95.84 |
| 80 | 96 | 180 | 86.93 |
| 60 | 0 | 180 | 80.38 |
| 60 | 20 | 180 | 88.17 |
| 60 | 40 | 180 | 90.41 |
| 60 | 60 | 180 | 92.24 |
| 60 | 80 | 180 | 93.93 |
| 60 | 96 | 180 | 92.12 |
| 60 | 96 | 60 | 91.73 |
| 60 | 96 | 120 | 93.37 |
| 60 | 96 | 180 | 89.53 |
| 60 | 96 | 240 | 92.46 |
| 60 | 96 | 300 | 89.67 |
| 60 | 96 | 360 | 82.67 |
